# The Current Status of Clinical Research Involving Microneedles: A Systematic Review

**DOI:** 10.3390/pharmaceutics12111113

**Published:** 2020-11-19

**Authors:** Seung-Yeon Jeong, Jung-Hwan Park, Ye-Seul Lee, Youn-Sub Kim, Ji-Yeun Park, Song-Yi Kim

**Affiliations:** 1 Department of Anatomy and Acupoint, College of Korean Medicine, Gachon University, Seongnam 13120, Korea; tmddus123x@gachon.ac.kr (S.-Y.J.); yeseulee@gachon.ac.kr (Y.-S.L.); ysk@gachon.ac.kr (Y.-S.K.); 2 Department of BioNano Technology and Gachon BioNano Research Institute, Gachon University, Seongnam 13120, Korea; pa90201@gachon.ac.kr; 3 College of Korean Medicine, Daejeon University, Daejeon 34520, Korea; jypark@dju.kr

**Keywords:** microneedle, systematic review, randomized controlled trial, research and development

## Abstract

In recent years, a number of clinical trials have been published on the efficacy and safety of drug delivery using microneedles (MNs). This review aims to systematically summarize and analyze the current evidence including the clinical effect and safety of MNs. Three electronic databases, including PubMed, were used to search the literature for randomized controlled trials (RCTs) and clinical controlled trials (CCTs) that evaluated the therapeutic efficacy of MNs from their inception to 28 June 2018. Data were extracted according to the characteristics of study subjects; disorder, types, and details of the intervention (MNs) and control groups; outcome measurements; effectiveness; and incidence of adverse events (AEs). Overall, 31 RCTs and seven CCTs met the inclusion criteria. Although MNs were commonly used in skin-related studies, evaluating the effects of MNs was difficult because many studies did not provide adequate comparison values between groups. For osteoporosis treatment, vaccine, and insulin delivery studies, MNs were comparable to or more effective than the gold standard. Regarding the safety of MNs, most AEs reported in each study were minor (grade 1 or 2). A well-designed RCT is necessary to clearly evaluate the effectiveness of MNs in the future.

## 1. Introduction

Microneedles (MNs) are therapeutic devices that consist of a single or an array of micrometer-sized needles that efficiently deliver drug components to the outer layers of the skin [1]. The needle length ranges from 25 to 1000 μm, and this is sufficient to cross the stratum corneum barrier and to reach in the dermis [2]. The microscopic length of MNs has the advantage of reducing the vasovagal reaction, stress or anxiety caused by needle phobia [3,4], and pain that occurs when a conventional needle is used [5]. MNs are manufactured using various materials such as silicon, metal, and glass. The shape of the needles can vary from cones to square pyramids, depending on the manufacturing method [6]. MNs can be classified into five typical forms, such as solid MNs, coated MNs, dissolving MNs, hollow MNs, and hydrogel-forming MNs, depending on the desired method of drug delivery [7]. Solid MNs enable the formation of temporary microchannels through the stratum corneum of the skin to increase the absorption of various drug formulations including creams, gels, solutions, and transdermal patches [8,9]. Coated MNs provide a method for coating a drug formulation onto a needle so that the drug is absorbed by the skin when the needle is inserted [10]. Dissolving MNs contain a drug that dissolves and is released over time when it contacts the interstitial fluid after being inserted into the skin [11]. Hollow MNs deliver a specific drug to the skin through an inserted hollow needle and may continuously deliver the drug by applying pressure to the formed channels or by generating an electrical flow [12]. Finally, hydrogel-forming MNs do not contain drugs in the needles, similar to coated or dissolving MNs, but adhere to the skin, causing a swelling action from the tip of the MNs. A drug contained in the attached reservoir layer of the MNs may then diffuse throughout the body by micro-recirculation of the skin [13].

The MN devices that are being used in the clinical practice include the microneedle therapy system (MTS), microneedle radiofrequency (MRF), hollow MNs, and microneedle array patches (MAPs). The MTS uses MN-embedded rollers or stamps and includes an auto-MTS (AMTS) as an automated improvement [14,15,16]. The MTS is primarily used for medical and cosmetic issues that occur in the skin, such as acne scars [17], stretch marks, and wrinkles [18]. The mechanism is based on neo-angiogenesis and neo-collagenesis processes caused by mechanical stimulation of the natural wound-healing response [19]. MRF is a device that combines fractional radiofrequency (RF) and MTS technology to directly deliver thermal energy with minimal invasion into the dermis [20]. This has been used for skin rejuvenation [21], face lifts [22], and axillary hyperhidrosis [23,24]. While MTS and MRF are commonly used locally on the skin, hollow MN fabrication and characterization have been extensively studied for systemic drug delivery [25]. As an alternative to the conventional needles, hollow MNs have been used for influenza vaccine delivery [26] and insulin delivery for diabetic patients [27,28]. Lastly, MAPs have been developed by combining transdermal patches with the MNs’ ability to deliver various molecules [29]. This has the advantages of convenience and safety. The MAPs have typically been designed as coated or dissolving MNs [30]. Recent studies have described coated MAPs being used in clinical trials for glucagon delivery to treat hypoglycemia, parathyroid hormone (PIH) to treat osteoporosis, and zolmitriptan to treat migraines [30,31]. In addition, dissolving MAPs have been applied in influenza vaccination studies and PIH delivery studies [32].

Previous reviews of MNs have addressed material safety [33,34], fabrication techniques [35], design and performance [36,37], drug delivery or skin irritation mechanisms [38], drug delivery methods [38], various applications including biosensor [39], gene guns [36], ultrasound [40], sonophoresis [41], and commercially available MNs [42]. These reviews have enhanced our understanding of the mechanical properties of MNs, the mechanism and basis for MNs utilization, and design and material selection used in MNs manufacturing. Studies on the clinical use of MNs are also increasing. Recently, a number of applications of MNs for the effective treatment of scars and wrinkles have been described [43]. In addition, some narrative reviews have been conducted and showed that delivery of various drugs including vaccines, insulin, and proteins could be done more effectively through MNs. However, few reviews exist that have systematically searched and analyzed the clinical value of MNs without limiting the types of diseases [34,35]. Therefore, this review aims to summarize and analyze the current evidence, including the clinical use and safety of MNs, and to explore the feasibility of using MNs for future medical applications.

## 2. Methods

### 2.1. Search Strategy

Electronic searches were conducted from their inception to 28 June 2018 without language restrictions using PubMed, the Research Information Sharing Service, and the National Digital Science Library. The search terms used were “microneedle”, “micro-needle”, “nanopatch”, “nano patch”, “micro patch”, “micro band”, “nano needle”, “micro therapy”, and “microneedle therapy”, with minor adjustments for each database. Furthermore, our search strategy included screening the reference lists of articles found in our original search strategy.

### 2.2. Study Selection

We included randomized controlled trials (RCTs) or clinical controlled trials (CCTs) that evaluated the therapeutic efficacy of MNs as compared with controls, regardless of disease types. First, we screened and excluded studies as follows:

- Studies not related to MNs.
- Studies related to MNs, but not to therapeutics (e.g., studies on the manufacturing and production of MNs and studies involving disease diagnosis using MNs).
- Nonclinical studies (e.g., studies of preclinical trials including animal studies, reviews, protocols, letters to editors, or expert opinions).

In addition, we excluded studies that were difficult to evaluate the therapeutic effect of MNs (i.e., no appropriate controls were used, or MNs were used as co-intervention). Due to language limitations, this review only included articles in both English and Korean. During the selection process, the inclusion and exclusion criteria were selected based on the title, abstract, and full text, if necessary. There was no limitation on study year, or country. Two researchers independently reviewed the papers, and discrepancies were resolved through discussion and consensus.

### 2.3. Data Extraction and Analysis

Data for the selected studies were extracted as follows: type of study design, characteristics of study subjects, disorder, types and details of the intervention and control groups, outcome measurements, effectiveness, and adverse events (AEs). MN types were classified as MTS, MRF, hollow MNs, and MAP, and the results from each study were extracted based on the comparison values between groups according to the data described in each paper.

To analyze the included studies, they were initially classified according to disease types, and then the results were analyzed for the effect of MNs as compared with a control group for each disease. In addition, the details of the MNs used in the included studies were reviewed to confirm how the MNs were used in the clinical studies. For the intervention group, the method of using MNs was divided into cases in which MNs were used alone, MNs used with drugs to facilitate drug delivery, or MNs used to improve the treatment effect of conventional devices, in addition to device types, including MTS, MRF, hollow MN, and MAP. The numbers and types of AEs reported in the included studies were summarized and analyzed according to MN type and disorder. The AEs reported in each study were categorized as either local or systemic, according to the treatment and control groups. In addition, the severity of the AEs was evaluated in grades from 1 to 5, according to the modified Common Terminology Criteria for Adverse Events, version 5.0 (Supplementary material Table S1) [44].

### 2.4. Risk of Bias Assessment

Study quality was assessed using the Cochrane risk of bias (RoB) tool [45]. This tool consists of six domains (selection bias, performance bias, detection bias, attrition bias, reporting bias, and other bias). The CCTs were assessed by the modified version of the RoB tool in which two domains related to selection bias (bias in the selection of participants into the study and bias due to confounding) were modified by referring to the ROBINS-I tool [46], a RoB tool for evaluating nonrandomized studies of intervention. One independent investigator used the RoB tool to evaluate study quality, while another investigator reviewed the results. Using this process, any controversy raised during the review process was resolved through discussion. The analyzed RoBs were reported using the Cochrane tool of the Review Manager 5.3 program (Cochrane, London, UK, 2014).

## 3. Results

From the screening based on the title, abstract, and full text if necessary, 5624 articles were selected from a total of 6166 through our database search after deleting 542 duplicate records. Among these, studies not related to MNs (*n* = 4388); studies related to MNs, but not to therapeutics (*n* = 643); and nonclinical studies (*n* = 496) were excluded in the first round of screening. The remaining articles were excluded through eligibility evaluation if they were not published in English or Korean (*n* = 3), were not studies analyzing the effectiveness of MNs (i.e., MNs were used as co-intervention) (*n* = 4), or were not controlled studies (*n* = 52).

**Figure 1:**
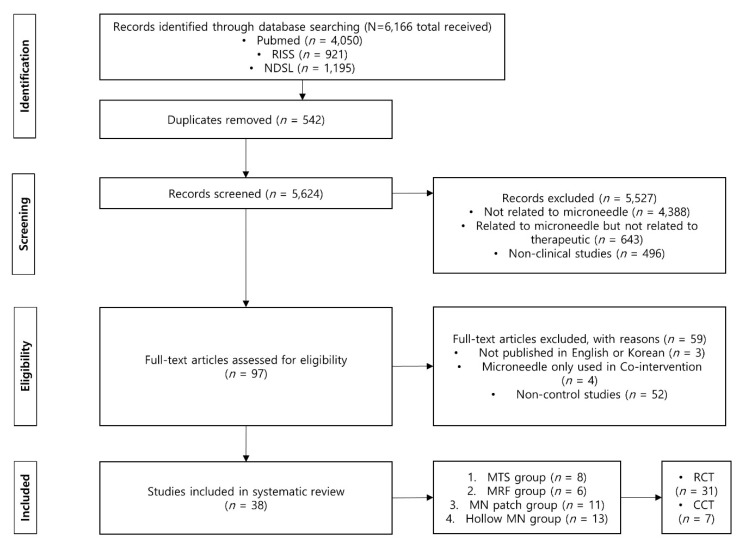
Flow chart for selection of studies.

Finally, 38 studies (31 RCTs and 7 CCTs) that met our inclusion criteria were selected (Figure 1). Among the studies, 19 studies were cross-over design studies in which one subject received both treatments at intervals or studies in which one subject was provided with two treatments at different sites simultaneously. In all cross-over design studies, only the first session was analyzed. The average sample size for the RCTs included in the review was 165.7 ± 319.7, whereas that of the CCT was 18.8 ± 7.8.

Twelve studies were conducted in the USA and Korea; three in Israel; two in China; and one in Australia, Bangladesh, Belgium, Egypt, France, Germany, Iran, Japan, and Switzerland (Supplementary material Figure S1). All studies were written in English except for two articles written in Korean.

**Figure 2:**
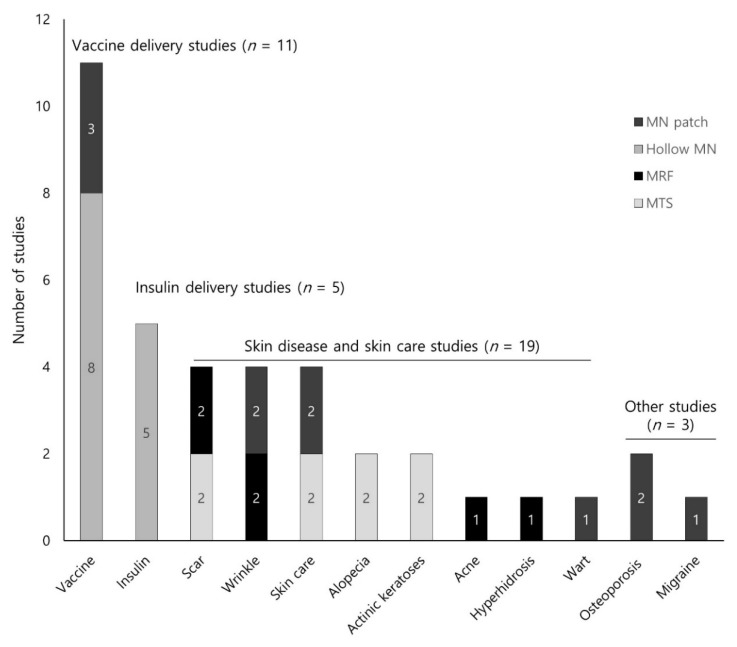
Number of studies included by diseases or microneedles types.

In this review, the most frequent type of MNs discussed was hollow MNs (*n* = 13), followed by MAP (*n* = 11), MTS (*n* = 8), and MRF (*n* = 6) (Table 1). We placed the diseases described in the studies into the following four categories: (1) skin diseases and skin care (*n* = 19, 50.0%); (2) vaccine delivery (*n* = 11, 28.9%); (3) insulin delivery for the treatment of diabetes (*n* = 5, 13.2%); and (4) others (*n* = 3, 7.9%, two osteoporosis and one migraine). Supplementary material Table S2 summarizes the details of each study based on disease. According to disease, MTS (*n* = 8, 88.9%), MRF (*n* = 6, 100%), and MAP (*n* = 5, 45.5%) were most frequently used in skin-related studies, whereas hollow MNs (*n* = 8, 72.7%) was primarily used in vaccine delivery studies (Figure 2).

### 3.1. Effectiveness: Outcomes of the Included Studies

#### 3.1.1. Skin Diseases and Skin Care (*n* = 19)

Scars, wrinkles, skin condition (*n* = 4 respectively, 21.1%); alopecia, actinic keratosis (AK, *n* = 2 respectively, 10.5%); and hyperhidrosis, acne, and warts (*n* = 1 respectively, 5.3%) were included in skin diseases and skin care studies. MTS (*n* = 8), MRF (*n* = 6), and MAP (*n* = 5) were used for drug delivery.

##### Scar (*n* = 4)

For the treatment of scars, each study compared MTS or MRF with laser therapy (CO_2_ laser or Erbium-glass fractional laser) compared with no treatment control. In the evaluation of both physicians and patients, the MTS was observed to be superior to CO_2_ laser for scar treatment [50]. However, other studies did not provide intergroup comparison results for the measurement of outcome [53,56,57].

##### Facial Wrinkle (*n* = 4)

In each study, MRF or MAP was topically applied to reduce facial wrinkles including periorbital and/or nasolabial wrinkles. The data did not clearly show that MRF was superior to botulinum toxin A injection [55] or superficial skin insertion [58]. Different results were observed for various outcome measures related to skin wrinkles when evaluated at various time points. Regarding wrinkle improvement, MAP showed no difference at four and eight weeks as compared with the placebo patch; however, at the end of the treatment (12 weeks), there was a significant difference between the two groups [61]. In a study using wrinkle cream as a control group, there was some evidence showing that the additional use of MAP with wrinkle cream was more effective than using wrinkle cream alone [64].

##### Skin Care (*n* = 4)

In a placebo-controlled study for facial hyperpigmentation, the efficacy of MAP was confirmed for some time points [60]. MAP was more effective in skin brightness and melanin index as compared with whitening essence [63]. Two studies using MTS reported only pre- and posttreatment comparison data instead of a comparison between the groups [47,49].

##### Alopecia (*n* = 2)

In two studies on alopecia, it was observed that an additional application of MNs (MTS or AMTS) was more effective for drug delivery. The additional use of AMTS for minoxidil treatment showed positive results in hair density and growth as compared with minoxidil treatment alone [48]. A study using methyl 5-aminolevulinic acid cream with MTS did not provide comparisons between groups [14].

##### Actinic Keratosis (*n* = 2)

For AK, the MTS with aminolevulinic acid (ALA) application yielded a positive effect in the evaluation of transepidermal water loss as compared with a sham roller [52]. In another study, the addition of MTS to ALA alleviated the symptoms of AK perceived by patients as compared with the application of ALA alone, but there was no difference between groups according to the physician [51].

##### Other Skin Type Studies (*n* = 3)

Other skin-related studies included acne, hyperhidrosis, and warts. MRF was effectively observed for hyperhidrosis disease severity scale and visual analogue scale (VAS) of sweating intensity score compared with sham treatment of hyperhidrosis [23]. However, in the acne study, there was no statistically significant difference and no described statistical comparison with the CO_2_ laser [54].

#### 3.1.2. Vaccine Delivery (*n* = 11)

For vaccine delivery studies, influenza (*n* = 7), poliovirus (*n* = 2), and rabies (*n* = 2) vaccines were delivered through MNs. These studies evaluated whether the intradermal (ID) injection method using MNs showed a similar immunogenicity at a small dose compared with the conventional injection methods. Influenza vaccine delivery studies included three MAP studies and four hollow MNs studies. For influenza vaccine delivery studies, delivery with MNs in small doses was better or similar in geometric mean titers (GMT) for antibody titers, seroconversion, and seroprotection compared with intramuscular (IM) injection [26,64,65,68,71], subcutaneous (SC) injection [32], or Mantoux [70,72], which is a traditional ID delivery method. For poliovirus vaccine delivery studies, hollow MNs with small doses was similar in GMT for antibody titer, seroprotection [74], seroconversion for humoral immunogenicity, and intestinal mucosal immunity [73] compared with IM. For rabies vaccine delivery studies, hollow MNs with small doses was superior in GMT for antibody titer, seroconversion, and seroprotection compared with epidermal injection and topical applications [68], and exhibited similar effects compared with an ID injection using Mantoux [78] and IM [68,78].

#### 3.1.3. Insulin Delivery (*n* = 5)

All studies used hollow MNs for insulin delivery. In an insulin delivery study for type 1 diabetes, hollow MNs provided faster absorption through faster onset and offset compared with SC [69,71]. Hollow MNs was also superior in postprandial glucose control [69,71,75], insulin availability through *C*_max_ and *T*_max_ [75], intrasubject variability, and intersubject variability for *T*_max_ compared with SC [27,75]. In type 2 diabetes studies, hollow MNs resulted in faster absorption through faster onset and better postprandial glucose control compared with SC [77].

#### 3.1.4. Others (*n* = 3)

In osteoporosis studies, 40 μg PIH [31] and 40 μg teriparatide (TPTD) [59] were used. These studies evaluated whether the ID injection method using MNs showed similar immunogenicity at a small dose compared with the conventional injection method. A MAP with 40 μg PIH and 40 μg TPTD was superior in the evaluation of hip bone mineral density (BMD) compared with the injection pen and placebo patch. MAP was more effective for the lumbar spine BMD compared with the placebo patch. In a migraine study, MAP showed more effectiveness on headache reliefs compared with the placebo patch as the zolmitriptan dose increased [67]. In each study, other indicators did not exhibit statistically significant differences.

### 3.2. Safety: Adverse Events and Microneedle-Induced Pain

#### 3.2.1. Adverse Events

Of the selected studies, three did not have information regarding AEs [47,51,52,61] and two studies reported that there were no AEs in either group [63,64]. Of the 32 studies reporting AEs, all AEs from studies using MTS and MRF (*n* = 12) were grade 1 (mild grade). All grade 1 AEs were resolved during the study period. Of the MAP studies, grade 2 AEs were reported in two studies for which local or noninvasive interventions were provided. These AEs were all resolved during the study period [59,67]. Of the hollow MNs studies, two reported grade 3 AEs [70,74] and one study reported grade 5 AEs [69]. A grade 3 AE in the influenza vaccine delivery study led to a hospitalization and was considered to be related to the vaccine [70]. In the poliovirus vaccine delivery study, five grade 3 AEs and three grade 5 deaths were reported, but they were unrelated to the MNs or vaccine (Supplementary material Table S3) [74].

#### 3.2.2. Microneedle-Induced Pain

MNs-induced pain was reported in 18 studies (MTS, *n* = 3; MRF, *n* = 3; MAP, *n* = 3; hollow MNs, *n* = 9), and pain severity was measured by VAS in 14 studies. One study was evaluated through a questionnaire using a scale of 1 (negative experience) to 5 (positive experience). In four studies, the severity of pain was not described (Table 2). In studies involving MTS (*n* = 3), MTS showed statistically higher pain compared with a sham roller [52]. In the other two studies, pain was reported only in the treatment group [14,48]. Of the three studies reporting MRF-induced pain, one did not report pain in the control group (CO_2_ laser), and two reported pain in the control group (CO_2_ laser, BoNT/A), but there were no significant differences between the groups. The MAP exhibited a statistically low pain (VAS) value compared with cryotherapy [65]. MAP-induced pain was significantly less than that of the control group. There were nine hollow MNs studies that performed pain analysis, and six of them assessed a MNs-induced pain index by dividing it into insertion and infusion pain. Of the six studies, five involving hollow MNs exhibited significantly lower insertion pain and higher infusion pain compared with ID [78], SC [27,71,77], and IM [26,78]. One study reported higher insertion and infusion pain compared with SC [75]. In three other studies, hollow MNs resulted in higher pain compared with SC [69] or IM [70], and exhibited lower pain compared with IM and ED [68], although there was no statistical comparison.

### 3.3. Qualitative Assessments

#### Risk of Bias in Randomized Controlled Trials (*n* = 31)

**Figure 3:**
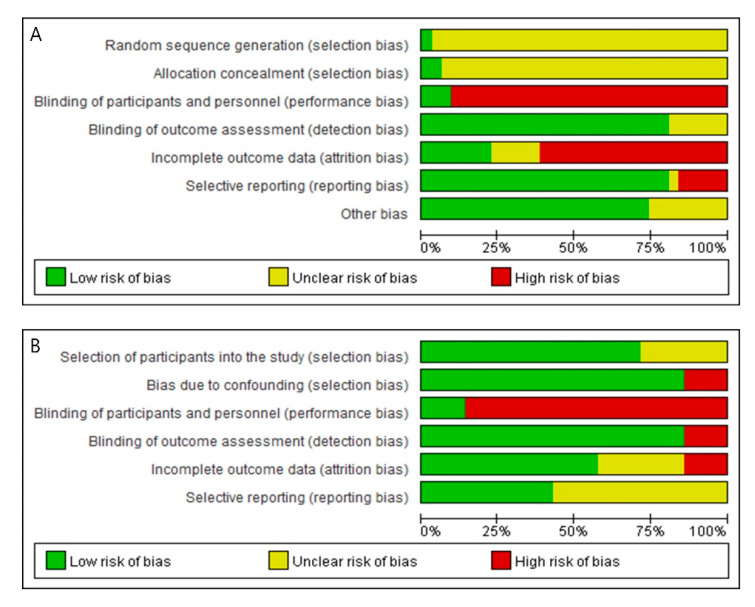
Risk of bias graph: (**A**) Risk of bias graph of randomized controlled trials (RCTs); (**B**) Risk of bias graph of controlled clinical trials (CCTs).3.3.2. Risk of Bias in Clinical Controlled Trials (Non Randomized Studies) (*n* = 7).

In the evaluation of the RoB in the final 31 randomized studies, none satisfied all seven bias domains (Figure 3A, Supplementary material Figure S2). Except for two studies [59,62], the information necessary to evaluate RoB was not described for two domains to evaluate selection bias, including a random sequence generation method and a specific method related to allocation concealment. A high risk of performance bias was observed, and it was confirmed that only three studies were clearly double-blinded [52,60,67]. Since the control group of the included study was considered a gold standard and not a placebo control, it was apparent that the blinding of subjects and researchers was impossible. In the case of detection bias, the RoB was relatively low (*n* = 25, 80.6%: low RoB). The risk of attrition bias was relatively high. Except for the eight studies (25.8%) in which all subjects completed the study, no information was provided on whether there was missing data (*n* = 5), and even if there was missing data, the values were not handled using an appropriate statistical method (*n* = 18). Risk of reporting bias due to selective reporting was relatively low (*n* = 25, 80.6%: low RoB). We could not identify a pre published protocol study to confirm selective reporting. In the evaluation of bias based on the omission of the results described in the methods, it was confirmed that the risk of reporting bias was high in six studies. In addition, we analyzed the balance of the major variables before treatment between the treatment and control groups to examine the presence of other biases. As a result, most of the studies (*n* = 23, 74.2%) showed no differences between the baseline groups.

Risk of bias analysis of 7 non-random studies was modified by referring to ROBINS-I tool for two selection biases, none of the studies satisfies all 6 bias domains (Figure 3B, Supplementary material Figure S3). Bias in selection of participants into the study analyzed whether subjects were selected from the same population. As a result, five studies were set to “low risk” by stimulating two groups in each different site to one subject, and two studies were set to “unclear risk” because there was no content on the difference between the two groups [50,56]. Bias due to confounding was analyzed through the presence or absence of confounding variables affecting the results. In the scar study, the subject’s scar classification was classified into two, and it was classified as “high risk” because it did not proceed within the same scar type [50], and six other studies were classified as “low risk” because the age, sex and skin type were matched. As a result of the blinding of participants and personnel analysis, all six except for one double-blinded study [61] were classified as “high risk” because the MNs and the control group could be distinguished. In the case of blinding of outcome assessment, the subject’s application position was already determined in the analysis study using photographs, and because it was not an objective analysis, it was classified in one study as “high risk” [55] while it was classified as “low risk” in all of the other studies. As a result of the analysis of incomplete outcome data, one study with missing values was “high risk” [55], two studies without mention of missing values were “unclear risk” [48,61], and the remaining 4 studies were “low risk”. As a result of the selective reporting analysis, four studies that analyzed AE but did not analyze the difference between the treatment group and the control group were classified as “unclear risk” [23,48,56,61], and other studies were classified as “low risk”.

## 4. Discussion

This study was conducted to systematically summarize and analyze current evidence, including the clinical effect and safety of MNs, which have recently become an important research field for transdermal drug delivery. Overall, 38 studies (RCT, 31; CCT, 7) were analyzed to 28 June 2018. Four types of MNs (MTS, 8; MRF, 6; MAP, 11; hollow MNs, 13) were described according to application methods that have been conducted for cosmetic (i.e., facial skin condition, such as wrinkle, hyperpigmentation, skin brightening) or medical purposes. MNs have been used in the treatment of skin diseases (i.e., scar, wrinkle, alopecia, AK, wart), delivery of specific drugs (i.e., vaccine or insulin delivery), and for other medical purposes (i.e., osteoporosis, migraine, axillary hyperhidrosis). Regarding vaccine delivery, insulin delivery, and migraine treatment, MNs showed similar or more effective results than control groups. In the case of skin-related and osteoporosis studies, MNs-mediated drug delivery was more effective than drug application only or placebo MNs. Overall, the effect of MNs reviewed in this study seemed to be higher than that of the conventional devices. However, many indicators have not been adequately compared between groups, and further studies are required for a conclusive analysis. Regarding the safety of MNs, most AEs reported in each study were minor (grade 1 or 2). Serious AEs of grade 4 or higher were not included among the AEs related to MNs in these studies.

One important characteristic of this study compared to previously published reviews is the exploration of the values of MNs based on clinical research. This study puts an emphasis on the evaluation of clinical effectiveness of MNs based on clinical studies. In the course of development of MNs, research on technical problems and pharmacological issues related to drug delivery were considered very important [36,38,41]. Some of the examples of technical problems raised during the development of MNs systems include design, mechanical testing, skin penetration and insertion force, MNs uniformity, sterilization, stability, and biocompatibility, all of which have been dealt with in the previous studies [79]. Other reviews related to the clinical application of MNs have been reported for scars and wrinkles [43,80], vaccines, insulin, and protein delivery [34,35]. While previous studies emphasized the importance of the management based on the safety and efficacy of MNs, many of these previous studies focused on the technological advances of individual MNs or the treatment of individual diseases [81]. A systematic literature reviews involving MNs based on RCTs or CCTs have been rarely performed. In order to address these issues, we compared and analyzed the efficacy and safety of MNs from studies evaluating MNs in the treatment and control groups.

In this systematic review, we observed that MNs have been used in the treatment of a wide variety of diseases. In skin-related studies, MNs have been used to treat scars and wrinkles and for cosmetic skin care. In the 19 studies examined, MNs showed more effective results compared with the control group in each study. The one exception was a wrinkle study by Lu [58] in which a superficial dermal insertion showed more effective results compared with MNs for the clinical assessment of infraorbital wrinkles. However, many outcome indicators lacked comparison values between groups, and integrating these results was difficult because of the use of various controls for each disease. In osteoporosis studies, the effectiveness of MNs was determined by showing similar or more effective results compared with the gold standard, SC [31,59]. However, there were indicators that did not report comparison values between groups. The migraine study showed the effect of higher doses of zolmitriptan compared with placebo [67]. In vaccine and insulin delivery studies, the superiority of MNs was analyzed by applying gold standards such as IM injection and ID delivery using Mantoux as controls. Compared to the conventional treatments, MNs are known to have advantages such as no requirement for specialized training to administer, reduced cost through the use of less vaccine [82], and circumvention of needle phobia [83], which was partially confirmed in this review. In particular, diabetes studies have been mainly focused on type 1 diabetes. The typical patients with type 1 diabetes are children and adolescents. In these cases, avoiding injections may be common because of needle phobia, and approximately 25% of these patients under-administer their medication [84,85]. Since the type 1 diabetes subjects included in this review were primarily adults, the benefits of MNs for children and adolescents are only estimable. In addition, as a result of the MNs-induced pain for vaccine and insulin delivery indicated that the insertion pain that occurred at the moment of MNs application was less compared with the control group. However, regarding infusion pain at the moment of drug delivery, pain induced from MNs was often higher. In this case, the development of technology to relieve the infusion pain caused by MNs is considered necessary because the continuous use of the needle may have a negative effect on subjects who show resistance to pain.

Most of the studies have described the safety of MNs (84.2%). Two studies did not develop AEs, and three studies had serious grade 3 or higher AEs. Among the grade 1 and 2 AEs that occurred in the MNs treatment group, the most common side effect was erythema, which appeared to be from skin irritation due to MNs. All of the AEs were resolved within the study period. These results indicate that, although MNs were a relatively safe treatment intervention, it can routinely cause minor local adverse reactions because of skin irritation. In the future, it will be necessary to develop MNs products with minor adverse reactions and to sufficiently inform patients about these events during the procedure.

Treatment group selection according to MNs type is specific according to the type of disease and purpose of use. MRF and MTS were used for scars and wrinkles, which are treated through the formation of skin collagen and wound healing. Most of the studies focused on topical drug delivery involved pretreatment using MTS and then drug application, and the topical drug delivery studies were delivered using a MAP or hollow MNs. The choice of control group for MNs studies was specific for each disease. When MRF was selected as a treatment group, the control group involved sham treatment without electrical stimulation through the device used for conventional skin treatment or the standby mode to analyze the effect of MNs with electrical stimulation [23]. For the MAP or hollow MNs used for vaccine and insulin delivery, gold standards, such as IM, SC, and ID, were defined as controls, or the placebo control [61,62,86,87] of the drug itself, or a single MNs that did not contain a drug [60]. For MTS without drug delivery through the device itself, there was a study in which a roller without a needle was established as a sham roller [52], but this may have caused bias because the subject could detect skin invasion. There was no sham-type control group using an MNs stamp, and this review was analyzed in comparison with the drug application [14,51] or another treatment [88]. For the continuous expansion of MTS’s skin cosmetic market and drug delivery research and development, sham-type devices are required to analyze the specific effects of MNs on skin irritation [89].

Regarding the RoB analysis in RCTs, for random sequence generation, all studies mentioned randomization, without any description on the method except for one study. Allocation concealment analysis revealed that there was no allocation method in all but two studies. For future RCT studies, it is necessary to include detailed explanations to estimate low selection bias. In most of the studies, a performance bias corresponding to “high risk” occurred, and this was a problem caused by the difference between the appearance of MNs and control. Therefore, new devices and research to solve this problem are also needed.

This study presents the following implications along with the recent trends in MNs research & development. In this review, the clinical application of MNs was divided into the fields of beauty and medicine, in fact, the large industrial market of MNs is similar. Cosmetics are developed and sold such as MAP or roller type MNs for the purpose of wrinkle improvement, acne improvement, whitening, etc. Similarly, the RCTs and CCTs included in this review were also studied for a scar, wrinkle, or hyperpigmentation. MNs devices that deliver drugs through pores formed by MNs are used mainly for cosmetic purposes or local skin stimulation because the amount of drug delivered is extremely small, and many products of these types of MNs are already available on the market. On the other hand, MNs devices within the pharmaceutical and medical device industries require more complex regulations and considerations than the beauty industry [81]. In this case, since an effective drug must be delivered at a significant dose, the product can only be released by passing through the development pipeline, from phase 1 to U.S. Food and Drug Administration (FDA) approval, evaluating safety, efficacy, and effectiveness. In addition to RAPHAS in Korea, companies such as Quadmedicine, 3M, Corium, and Zosano Pharma have developed drugs or vaccines for dementia, osteoporosis, cancer, and migraine in the form of MNs, and they are conducting phase 2 and 3 clinical studies [90]. Recently, Zosano Pharma was approved by the FDA for a New Drug Application (NDA) review for MAP (Qtrypta™) for acute treatment of migraines [91]. Although we have not been able to analyze all of these rapidly changing and latest knowledge, in this review, MNs for influenza, rabies, poliovirus vaccine, insulin delivery, osteoporosis, and migraines were discussed. Vaccine studies analyzed non-inferiority compared with the gold standard control group each season. Insulin studies analyzed the faster response and persistence of MNs, whereas osteoporosis and migraine studies compared and analyzed the effective indicators of drug delivery using MNs.

In this review, it was confirmed that the research and development of MNs faced various challenges. Firstly, compared to the MNs industry, which is characterized by rapid growth, clinical research that applied MNs as a medical device was relatively slow in the process, because the research was conducted with approval for each item of MNs individually. Secondly, the advances made in the industry was not always reflected in the clinical studies. In the technical part of the MNs industry, there have been reports of a number of technologies that have applied clinical necessities such as material application [92], drug dose control technology using wireless magnetic [93], MNs application using 3D print [94], mass production using molding technology [95], a feedback system about attachment method, time, and administration for quantitative delivery of MNs [96]. These technologies have not been sufficiently applied in clinical studies. In addition to the minimal clinical studies required in the patent and licensing process, various population groups and clinical studies based on the real world need to be supported. Thirdly, there is a need for improvement and application in technical aspects of the development and manufacturing, including a method of controlling the exact dose for drug delivery, MNs’ efficient mass production process technology, and standardized good manufacturing practice (GMP) facilities for medical devices. In specific, in clinical situations requiring repeated regular use with a small dose (i.e., insulin injections in diabetic patients, multiple injections of vaccines), appropriate results can be obtained only by conducting clinical studies with more rigorous manufacturing management and administration training. These processes should be adequately described in the clinical research protocol. This can be addressed through development of proper guidelines for clinical research on MNs. Fourth, from the perspective of MNs’ users, careful consideration such as how to properly use MNs is required, which will be an important factor in maximizing the effectiveness of MNs and maintaining safety.

This study has several limitations. First, it is possible that some studies were missed because of the limited search strategy. Considering the high growth rate of the MNs market, if a larger number of databases were searched along with the gray literature, it is possible that more studies could have been included. Language restrictions in English and Korean may also have influenced these limitations. Second, due to the strictness of the inclusion criteria, the nature of various clinical studies related to MNs could not be covered. Of the 6166 searched papers, 1232 papers were focused on MNs, but only 38 were suitable for our inclusion criteria. There were many MNs studies in humans, but case reports, reviews, and nontreatment research represented the majority, rather than controlled clinical studies aimed at evaluating efficacy and safety. In addition, since this study aims to determine the therapeutic efficacy of MNs, studies on healthy subjects and efficacy studies on diagnosis and monitoring were excluded. There have also been many studies using MNs for disease diagnosis and monitoring. These include hollow MN for diagnosis of latent tuberculosis infection, glucose monitoring using hydrogel-forming MNs in diabetic patients, electrocardiography, eletromiography, and electroencephalography. Third, this systematic review included only studies up to 28 June 2018, so clinical studies of recent MNs were not included. Recently published clinical studies of MNs include an RCT for influenza vaccine using MAP [97] and a CCT for Keloids using dissolving MN [98]. Finally, pooling the effects of MNs was not possible because of problems of the heterogeneity issues. In each study, there were many cases where the primary endpoint was not specified; in particular, various types of MNs, control, treatment duration, and outcome measures were used in a number of studies investigating the same disease. Furthermore, there were cases in which studies often did not provide adequate comparison data between groups.

This systematic review confirms that various types of MNs are being studied for various diseases in the medical field, as well as for cosmetics. For diseases such as wrinkles, hyperpigmentation, alopecia, AK, influenza, rabies, poliovirus, diabetes, and migraine, MNs showed clinical value. They exhibited equivalent or improved treatment effects compared with existing treatment devices and injection methods. In addition, MNs were relatively safe and caused only mild adverse reactions. Considering the relatively low quality of the studies analyzed, well-designed RCTs are needed to provide a clear basis for the effectiveness of MNs in the future. In addition, it should be utilized to develop and commercialize various MNs products through a large number of clinical trials involving microneedle systems.

## Figures and Tables

**Table 1:** Summary of the intervention and control.

MNs Type	Intervention			Control				
	MNs	MNs + Drug	MNs + Other Treatment	Other Treatment + Drug	Drug Application Only	Other Treatment Only	No-Treatment	Placebo
MTS (*n* = 8)								
Kim, 2009 [47]	**√**	-	-	-	**√**	-	**√**	-
Yoo, 2010 [48]	-	**√**	-	-	**√**	-	-	-
Choi, 2011 [49]	-	**√**	-	**√**	-	-	-	-
Khater, 2016 [50]	**√**	-	-	-	-	CO_2_ laser	-	-
Spencer, 2016 [51]		**√**			**√**			
Petukhova, 2017 [52]	-	**√**	-	-	-	-	-	**√**
Bao, 2017 [14]	**√**	**√**	-	-	**√**	-	-	-
Busch, 2018 [53]	-	**√**	-	-	-	-	**√**	-
MRF (*n* = 6)								
Shin, 2012 [54]	**√**	-	-	-	-	CO_2_ laser	-	-
Jeon, 2013 [55]	**√**	-	-	**√**	-	-	-	-
Ryu, 2013 [56]	**√**	-	CO_2_ laser	-	-	CO_2_ laser	-	-
Chae, 2014 [57]	**√**	-	-	**√**	-	-	-	-
Fatemi Naeini, 2015 [23]	**√**	-	-	-	-	-	-	**√**
Lu, 2017 [58]	**√**	-	-	-	-	Superficial dermal insertion	-	-
MAP (*n* = 11)								
Cosman, 2010 [59]		**√**		**√**				**√**
Daddona, 2011 [31]	-	**√**	-	**√**	-	-	-	**√**
Hirobe, 2015 [32]	-	**√**	-	**√**	-	-	-	-
Kim, 2016 [60]	-	**√**	-	-	-	-	-	**√**
Lee, 2016 [61]	-	**√**	-	**√**	-	-	-	-
Rouphael, 2017 [62]	-	**√**	-	**√**	-	-	-	**√**
Park, 2017 [63]	-	**√**	-	-	**√**	-	-	-
Hong, 2018 [64]	**√**	**√**	-	-	**√**	-	-	-
Ryu, 2018 [65]	-	**√**	-	-	-	Cryotherapy	-	-
Fernando, 2018 [66]	-	**√**	-	**√**	-	Intramuscularly injection	-	**√**
Spierings, 2018 [67]		**√**						**√**
Hollow MNs (*n* = 13)								
Damme, 2009 [26]	-	**√**	-	**√**	-	-	-	-
Laurent, 2010 [68]	-	**√**	-	**√**	-	-	-	-
Pettis, 2011 [69]	-	**√**	-	**√**	-	-	-	-
Frenck, 2011 [70]		**√**		**√**				
McVey, 2012 [27]	-	**√**	-	**√**	-	-	-	**√**
Norman, 2013 [71]	-	**√**	-	**√**	-	-	-	-
Levin, 2014 [72]	-	**√**	-	**√**	-	-	-	-
Anand, 2015 [73]	-	**√**	-	**√**	-	-	-	-
Troy, 2015 [74]	-	**√**	-	**√**	-	-	-	-
Rini, 2015 [75]	-	**√**	-	**√**	-	-	-	-
Levin, 2016 [76]	-	**√**	-	**√**	-	-	-	-
Kochba, 2016 [77]	-	**√**	-	**√**	-	-	-	-
Vescovo, 2017 [78]	-	**√**	-	**√**	-	-	-	-

-, that intervention was not used; **√**, that intervention was used; MAP, microneedle array patch; MN, microneedle; MRF, microneedle radiofrequency; MTS, microneedle therapy system.

**Table 2:** Intervention-induced pain of the included studies.

MNs Type	Intervention Group Pain: VAS		Control Group Pain: VAS [Type of Control]		*p* Value	
MTS (*n* = 3)						
Yoo, 2010 [48]	Pain (ND)		ND [MAL cream]		ND *	
Petukhova, 2017 [52]	1.3~1.4		0.3 [Sham roller]		*p* < 0.05 *	
Bao, 2017 [14]	4.52 ± 3.7		ND [Topical cream]		ND *	
MRF (*n* = 3)						
Shin, 2012 [54]	5.7 ± 1.7		6.5 ± 2.2 [CO_2_ laser]		NS	
Ryu, 2013 [56]	Pain (ND)		ND [CO_2_ laser]		ND	
Jeon, 2013 [55]	Pain (ND)		Pain (ND) [BoNT/A injection]		ND *	
MAP (*n* = 3)						
Rouphael, 2017 [62]	Pain (4%)		Pain (18%) [IM]		*p* < 0.05	
Ryu, 2018 [65]	0.5 ± 0.5		7.3 ± 1.3 [Cryotherapy]		*p* < 0.05	
Spierings, 2018 [67]	Pain (ND)		Pain (ND) [Placebo patch]		ND	
Hollow MNs (*n* = 9)						
	**Insertion**	**Infusion**	**Insertion**	**Infusion**	**Insertion**	**Infusion**
Damme, 2009 [26]	0.55	0.7	1.3 [IM]	0.6 [IM]	*p* < 0.05	*p* < 0.05*
Laurent, 2010 [68]	0~2		0~3.5 [IM], painless [ED]		ND	
Pettis, 2011 [69]	6.6 ± 3.9		4.1 ± 3.3 [SC]		ND	
Frenck, 2011 [70]	1.7~2.1		1.52 (IM), 2.2 [ID]		ND	
McVey, 2012 [27]	0.3	2.4	0.6 [SC]	1.1 [SC]	*p* < 0.05	*p* < 0.05 *
Norman, 2013 [71]	0.05~0.08	2~3	1.5~2 [SC]	0.05~1.5 [SC]	*p* < 0.05	NS *
Rini, 2015 [75]	Pain (ND)	Pain (ND)	Pain (ND) [SC]	Pain (ND) [SC]	ND *	
Kochba, 2016 [77]	0.9 ± 0.9	1.6 ± 1.5	0.7 ± 0.5 [SC]	0.4 ± 0.4 [SC]	NS*	*p* < 0.05 *
Vescovo, 2017 [78]	0~2	1~6	0~3 [ID], 0~4 [IM]	0~3 [ID], 0~4 [IM]	*p* < 0.05	*p* < 0.05 *

*, MNs site is more painful than control site; BoNT/A, botulinum toxin A; ED, epidermal injection; IM, intramuscular injection; MAL, methyl 5-aminolevulinic acid; MAP, microneedle array patch; MN, microneedle; MTS, microneedle therapy system; ND, no described; NS, no significant; SC, subcutaneous injection; VAS, visual analog scale.

## Supplementary Materials

The following are available online at https://www.mdpi.com/1999-4923/12/11/1113/s1, Figure S1: Number of studies included by country, Figure S2: Risk of bias summary of randomized controlled trials (RCT), Figure S3: Risk of bias summary of controlled clinical trials (CCTs), Table S1: Grade of adverse event by Common Terminology Criteria for Adverse Events (CTCAE), Table S2: Analysis of outcomes in included studies, Table S3: Type of adverse events reported in each study.
