# Supplementary material for: The Current Status of Clinical Research Involving Microneedles: A Systematic Review
Source: Pharmaceutics. 2020 Nov 19;12(11):1113. doi: 10.3390/pharmaceutics12111113 (PMC7699365; doi:10.3390/pharmaceutics12111113)
Supplement: Supplementary file 1 [file pharmaceutics-12-01113-s001.pdf]

pharmaceutics

MDPI

# Supplementary Materials: The Current Status of Clinical Research Involving Microneedles: A Systematic Review

**Seung-Yeon Jeong, Jung-Hwan Park, Ye-Seul Lee, Youn-Sub Kim, Ji-Yeun Park and Song-Yi Kim**

**Table S1.** Grade of adverse event by Common Terminology Criteria for Adverse Events (CTCAE).

| Grades | Clinical Descriptions |
|---|---|
| Grade 1 | Mild : asymptomatic or mild symptoms; intervention not indicated |
| Grade 2 | Moderate : minimal, local or noninvasive intervention indicated |
| Grade 3 | Severe but not immediately life-threatening : hospitalization indicated |
| Grade 4 | Life-threatening consequences : urgent intervention indicated |
| Grade 5 | Death related to adverse event |

This criteria is a modification of the Common Terminology Criteria for Adverse Events (CTCAE, Version 5.0) of department of health and human services (US).

**Table S2.** Analysis of outcomes in included studies.

| Author, Year, Country | Design | Participants Disorder, N (Int./Con.) | Intervention: Type of MNs | Control | Treatment Procedure: 1) site of MNs | Treatment Procedure: 2) N of Treatment (Duration, Frequency) | Outcome Measure | Results (Based on Between Groups Differences) |
|---|---|---|---|---|---|---|---|---|
| **Scar** | | | | | | | | |
| Khater, 2016, Egypt [1] | CCT | Striae distensae, 20(10/10) | MTS | CO2 laser | Int: Thighs, legs Con: Abdomen, lower limbs | 3 (3-mon, monthly) | [6-mon after final treatment] 1. Physician' assessment (0-4 scale); **2.** Patient satisfaction (0-4 scale); **3.** Biopsy | 1,2. $p < 0.001$; **3**. ND |
| Busch, 2018, Germany [2] | RCT* | Hypertrophic burn scar, 19(19/19) | A. MTS + vitamin, nourishing product | B. No treatment; C. No treatment (healthy skin) | A,B: Scar (randomly) C: Healthy skin | 5 (6-12-mon, ND) | [12-mon after final treatment] 1. POSAS ①Observer (thickness, pliability, and surface area) ② Patient (pruritus, thickness, relief, rigidity); 2. Skin moisture (Corneometer); 3. TEWL | 1~3. ND |
| Ryu, 2013, Korea [3] | CCT | Striae distensae, 30(10/10/10) | A. MRF; B. MRF + CO2 laser | C. CO2 laser | Abdomen, hips, or calves | 3 (3-mon, monthly) | [6-mon after final treatment] 1. Physician's global assessment (1-4 scale); 2. Patient satisfaction; 3. Biopsy | 1~3. ND |
| Chae, 2015, Korea [4] | RCT | Atrophic acne scar, 40(20/20) | MRF | Er:Glass fractional laser | Face (acne scars) | 3 (12-wk, 1/4-wk) | [8-wk after final treatment] 1. Patient satisfaction (1-5 scale); 2. Physician's global assessment (1-5 scale); 3. Scar severity (ECCA grading scale) | 1,2. ND; 3. NS |
| **Facial wrinkle** | | | | | | | | |
| Jeon, 2013, Korea [5] | CCT* | Wrinkles, 12(12/12) | MRF | BoNT/A injection | Periorbit (other side per group) | 3 (Int) (6-wk, 1/3-wk); 1 (con) | [At 3, 6, 18-wk] 1. Wrinkle grade (0-5 scale); 2. Skin elasticity (Cutometer); 3. Patient satisfaction (1-4 scale) | 1. $p < 0.05$ (Int<Con at 3,6-wk; Int>Con at 18-wk); 2. NS; 3. $p < 0.05$ (Int<Con at 3-wk), NS (at 18-wk) |
| Lu, 2017, China [6] | RCT* | Wrinkles, 13(13/13) | MRF | Superficial dermal insertion | Face (other side per group) | 3 (12-wk, 1/4-wk) | [12-mon after final treatment] 1. Clinical assessment (0-5 scale) ①Infraorbital wrinkle ②Nasolabial groove ③Overall improvement | 1. ①NS ②$p < 0.05$ (Int<Con) ③ND |
| Lee, 2016, Korea [7] | CCT* | Wrinkles, 23(23/23) | MAP | Placebo patch | Periorbit (other side per group) | 21 (12-wk, 1/4-day) | [At 4, 8, 12-wk] 1. GPS (0-7 scale); 2. Wrinkle seriousness (Visiometer) (R1. Skin roughness, R2. Maximum roughness, R3. Average roughness, R4. Smoothness depth, R5. Arthnetic average roughness) | 1. $p < 0.05$ (at 12-wk); 2. $p < 0.05$ (R1, R2, R3 at 12-wk) |

| | | | | | | | | |
|---|---|---|---|---|---|---|---|---|
| Hong, 2018, Korea [8] | RCT | Wrinkles, 84(27/27/30) | A. MAP; B. MAP + wrinkle cream | C. Wrinkle cream | Periorbit and nasolabial fold | 3 (12-wk, 1/4-wk) | [At 4, 8, 12-wk] 1. Wrinkle improvement ($R_a$ value) ①Crow's feet wrinkle ②Nasolabial fold; 2. Wrinkle grade (0-4 scale) ①Crow's feet wrinkle ②Nasolabial fold; 3. Skin roughness and wrinkle (Visiometer) (R1. Skin roughness, R2. Maximum roughness, R3. Average roughness, R4. Smoothness depth, R5. Arthnetic average roughness); 4. Skin hydration of eye rim (Cutometer, 0-4 scale); 5. TEWL (Vapometer) | [A vs. C] 1. ①,②NS; 2. ①, ②$p < 0.05$ (at 8-wk); 3. (R1~4. ND) R5. NS; 4,5. ND [B vs. C] 1. ①$p < 0.05$ (at 8-wk) ②NS; 2. ①$p < 0.05$ (at 4,8-wk) ②$p < 0.05$ (at 4-12-wk); 3. (R1~4. ND) R5. NS; 4,5. ND |
| **Skin care** | | | | | | | | |
| Kim, 2009, Korea [9] | RCT | Facial skin condition, 24(8/8/8) | A. MTS | B. AHA (7%); C. No treatment | Face | 6 (6-wk, weekly) | [At 6-wk] 1. Pore; 2. Large pore; 3. Blackhead; 4. Wrinkles; 5. Small pigmentation (number of 1~5); 6. Area of small pigmentation; 7. Number and area of large pigmentation; 8. Change of skin brightness; 9. Satisfaction (1~8, RSA) | 1~9. ND |
| Choi, 2011, Korea [10] | CCT | Facial skin condition, 14(7/7) | MTS + EGF | Treatment by high frequency + EGF | T, U-Zone of face | 6 (6-wk, weekly) | [2-wk after final treatment] 1. Skin oil; 2. Skin keratin; 3. Skin moisture; 4. Pore and wrinkle measurement; 5. Satisfaction (0~4, Aphrodite 3) | 1~5. ND |
| Kim, 2016, Korea [11] | RCT* | Facial hyperpigmentation, 45(A.23/23/B. 22/22) | MAP | Placebo patch | Int: face (left) Con: face (right) | A: 18 (8-wk, 1/3-day) B: 14 (8-wk, 1/4-day) | [At 4, 8-wk] 1. The skin depigmentation efficacy ①MI (Mexameter) ②ITA° value (Spectrophotometer) 2. Skin depigmentation (Images) | 1. ①A. $p < 0.05$ (at 4,8-wk), B. NS (at 4-wk) $p < 0.05$ (at 8-wk) ②A. NS (at 4-wk) $p < 0.05$ (at 8-wk), B. $p < 0.05$ (at 4-wk) NS (at 8-wk); 2. NS |
| Park, 2017, Korea [12] | RCT* | Skin brightening, 34(34/34) | MAP | Whitening essence | Cheek (other side per group) | 16 (8-wk, 2/weekly) | [At 2, 4, 8-wk] 1. Skin brightness (Chromameter); 2. MI change (Mexameter); 3. Skin whitening survey (0-4 scale) | 1. $p < 0.05$ (Int<Con at 4-wk; Int>Con at 8- wk); 2. $p < 0.05$ (at 2,4,8-wk); 3. ND |
| **Alopecia** | | | | | | | | |
| Yoo, 2010, Korea [13] | CCT* | Alopecia areata, 8(8/8) | MTS + MAL cream | MAL cream | Int: Scalp (right) Con: Scalp (left) | 3 (12-wk, 1/4-wk) | [1-mon after final treatment] 1. Hair regrowth (Photograph); 2. Histopathologic change (Microscopic) | 1,2. ND |

| | | | | | | | | |
|---|---|---|---|---|---|---|---|---|
| Bao, 2017, China [14] | RCT | Androgenetic alopecia, 60(20/20/20) | A. AMTS; B. AMTS + minoxidil | C. Minoxidil (topical) | Scalp | 12 (24-wk, 1/2-wk) | [At 24-wk] 1. Hair density ①The non-vellus hair ②The vellus hair ③The total hair; 2. Hair thickness (the non-vellus hair); 3. Hair growth ① Investigator assessment (7 point scale) ②Patient assessment (5 point scale); 4. Pain (VAS) | [A vs C] 1. ①NS ②$p < 0.05$ (C>A) ③NS; 2. NS; 3,4. ND [B vs C] 1. ①NS ②$p < 0.05$ ③$p < 0.05$; 2. NS; 3. $p < 0.001$; 4. ND |
| **Actinic Keratosis** | | | | | | | | |
| Petukhova, 2017, USA [15] | RCT* | Actinic keratosis, 32(A.16/B.16) | A. MTS + ALA (10-min) B. MTS + ALA (20-min) | C. Placebo roller | Face (other side per group) | 1 (1-mon, 1-day) | [At 1-mon] 1. AKs clearance (Photodynamic); 2. TEWL (Tewameter or Vapometer); 3. Pain (VAS); 4. Pain with blue light (VAS) | [A vs C] 1. NS; 2,3. $p < 0.05$; 4. NS [B vs C] 1. $p < 0.05$; 2,3. $p < 0.05$; 4. NS |
| Spencer, 2016, USA [16] | RCT* | Actinic keratosis, 20 (20/20) | MTS + ALA | ALA only | Face (other side per group) | 1 (4-mon, 1-day) | [At 4-mon] 1. Reduction in AKs; 2. Physician's global assessment (Visia camera image) | 1. $p < 0.05$; 2. ND |
| **Diseases of the skin (others)** | | | | | | | | |
| Shin, 2012, Korea [17] | RCT* | Acne, 20(20/20) | MRF | CO2 laser | Face (other side per group) | 1~2 (ND, ND) | [3-mon after final treatment] 1. Global improvement scale (0-4 scale); 2. The number of papules and pustules; 3. Satisfaction (0-3 scale); 4. Improvement sebum (Sebumeter); 5. Erythema index and pigmentation (Mexameter); 6. Pain (VAS) | 1~4,6. NS; 5. ND |
| Fatemi Naeini, 2015, Iran [18] | CCT* | Axillary hyperhidrosis, 25(25/25) | MRF | Placebo (standby mode) | Axilla (other side per group) | 3 (3-mon, 1/3-wk) | [At 3, 6, 9, 21-wk] 1. HDSS score (1-4 scale); 2. Sweating intensity score (VAS); 3. Patients satisfaction (6 point scale); 4. Histological evaluation | 1. $p < 0.01$ (at 6~21-wk); 2. $p < 0.001$ (at 3~21-wk); 3,4. ND |
| Ryu, 2018, Korea [19] | RCT* | Multiple warts , 42(42/42) | MAP | Cryotherapy | Wart (each lesion per group) | ND (until completely cleared, 1/2-wk) | [At 8, 16-wk] 1. The clearance rate; 2. Duration of treatment; 3. PGA and PaGA (0-5 scale); 4. Pain (VAS) | 1. ND; 2,3. NS; 4. $p < 0.0001$ |
| **Vaccine delivery** | | | | | | | | |
| Hirobe, 2015, Japan [20] | RCT | Influenza vaccine, 40(20/20) | MAP (a,b,c) | SC (a,b,c) | Upper arm (left) | 2 (3-wk, 1/3-wk) | [At 7, 21, 28, 42-days] 1. Immunogenicity (HI assay) in sera ①GMT ②Seroconversion ③ Seroprotection ④Increase in GMT ⑤Isotypes titer; 2. Immunogenicity (HI assay) in nasal wash ① | 1. ①~④ND ⑤NS; 2,3. ND |

| | | | | | | | | |
|---|---|---|---|---|---|---|---|---|
| | | | | | | | Increases in GMT ② Seroconversion ③Isotypes titer; 3. Counting of IFN-γ-producing cells (ELISPOT assay) (a. A/California/7/2009, b. A/Victoria/210/2009, c. B/Brisbane/60/2008) | |
| Rouphael, 2017, USA [21] | RCT | Influenza vaccine, 100(A.25/B.25/C.25/D.25) | A. MAP (a,b,c); B. MAP-self (a,b,c) | C. Placebo patch; D. IM (a,b,c) | A,C,D: wrist (non-dominant) B: Deltoid region | 1 (1-day, 1-day) | [At 28, 180-days] 1. Immunogenicity (HI assay) ① GMT ②Seroprotection③ Seroconversion; 2. Acceptability (1-5 scale) (a. A/Christchurch/16/2010, b. A/Texas/50/2012, c. B/Massachusetts/2/2012) | [A,B vs C] 1. ①NS ② $p < 0.01$ (a,c, A>C, B>C), NS (b) ③NS; 2. ND<br>[A,B vs D] 1. ①,②NS ③$p < 0.05$ (b. A>D, B>D); 2. $p < 0.05$ (28-day) |
| Fernando, 2018, Australia [22] | RCT | Influenza vaccine, 60(A.15/B.15/C.15/D.5/E.5/F.5) | A. MAP (FA, a,b,c); B. MAP (UA, a,b,c) | C. IM; D. Placebo MNs (FA, a,b,c); E. Placebo patch (UA, a,b,c); F. Placebo IM | A,D: volar forearm (FA) B,E: upper arm (UA) C,F: Deltoid region | 2) 1 (1-day, 1-day) | [At 3, 7, 21, 28-day] 1. Pain (VAS); 2. Acceptability; 3. Immunogenicity (HAI assay) ① GMT ②Seroprotection ③ Seroconversion; 4. Immunogenicity (Microneutralisation assay) ①GMT ②Seroprotection ③Seroconversion (a. A/California/07/2009, b. trivalent Fluvax® ) | [A,B vs C] 1,2. ND; 3. NS [A,B vs D,E,F] 1~3. ND |
| Damme, 2009, Belgium [23] | RCT | Influenza vaccine, 180(A.60/B.60/C.60) | A. Hollow MNs (3μg HA, a,b,c); B. Hollow MNs (6μg HA, a,b,c) | C. IM (15μg HA, a,b,c) | Deltoid region (non-dominant arm) | 1 (3-wk, 1-day) | [At 14, 21-day] 1. Pain (VAS) ①Insertion ② Infusion ③Overall; 2. Immunogenicity (HAI assay) ① GMT fold increas ② Seroconversion rate ③ Seroprotection rate (a. A/New Caledonia/20/99, b. A/Wisconsin/67/2005, c. B/Malaysia/2506/2004) | [A vs C] 1. ①,②$p < 0.05$ ③ NS; 2. NS<br>[B vs C] 1. ①$p < 0.05$ ②,③ NS; 2. NS |
| Laurent, 2010, France [24] | RCT | Rabies vaccine , 66(A.40/B.10/C.14/D.2) | A. Hollow MNs (a,b,c) | B. IM (a,b,c); C. ED (a,b,c); D. Topical (a,b,c) | Deltoid region (non-dominant arm) | 3 (21-day, 0,7,21-day) | [At 7, 14, 21, 49-day] 1. Immune response (RFFIT) ① GMT ②Seroconversion rate; 2. Pain (VAS) | [A vs B] 1. NS; 2. A<B ($p < 0.05$) [A vs C,D] 1,2. ND |
| Frenck, 2011, USA [25] | RCT | Influenza vaccine, 1592(A.400/B.399/C.395/D. | A. Hollow MNs (6μg HA, a,b,c); B. Hollow MNs | C. ID (Mantoux, 3μg HA, a,b,c); | A,B,C: Upper arm; D: Deltoid region | 1 (1-day, 1-day) | [At 21-day] 1. Immunogenicity (HAI assay) ① GMT ②Seroconversion rate ③ Seroprotection rate; 2. Pain (VAS) | [A vs C,D] 1,2. ND [B vs C,D] 1,2. ND |

| | | | | | | | | |
|---|---|---|---|---|---|---|---|---|
| | | 398) | (9μg HA, a,b,c) | D. IM (15μg HA, a,b,c) | | | (a. A/New Caledonia/20/99 IVR-116, H1N1, b. A/Wyoming/03/2003, H3N2, c. B/Jiangsu/10/2003) | |
| Levin, 2014, Israel [26] | RCT | Influenza vaccine, 280(A.56/B.56/C.56/D.56/E.56) | A. Hollow MNs (3μg HA, a,b,c) | B. ID (3μg HA, a,b,c); C. ID (4.5μg HA, a,b,c); D. ID (6μg HA, a,b,c)(ID: Mantoux); E. IM (15μg HA, a,b,c) | Deltoid region | 1 (1-day, 1-day) | [21-day after final treatment] 1. Immunogenicity (HI assay) ① GMT fold increase ② Seroconversion rate ③ Seroprotection rate (a. A/Solomon Islands/3/2006, b. A/Wisconsin/67/2005, c. B/Malaysia/2506/2004) | [A vs B] 1. ①$p < 0.05$ (a,c) ②$p < 0.05$ (b) ③$p < 0.05$ (c)<br>[A vs C] 1. NS<br>[A vs D] 1. ①$p < 0.05$ (a,c) ②$p < 0.05$ (strain c) ③NS<br>[A vs E] 1. ①$p < 0.05$ (b) ②, ③NS |
| Anand, 2015, Bangladesh [27] | RCT | Poliovirus vaccine, 922(A.203/B.200/C.156/D.152/E.211)(Type 1~3) | A. Hollow MNs (f-IPV, a,b,c); B. Hollow MNs (f-IPV/bOPV, a,b,c) | C. Oral (tOPV, a,b,c); D. Oral (bOPV, a,c); E. IM (IPV, a,b,c) | Thigh | A,E - 2 (14-wk, 6,14,-wk); B,C,D – 3 (14-wk, 6,10,14-wk) | [At 14, 18, 19-wk] 1. Humoral immunogenicity (CDC) ①Seroconversion by 14-wk ② Seroconversion by 18-wk; 2. Intestinal immunogenicity (CDC): Poliovirus shedding at 19-wk (a. Poliovirus type 1 (PV 1), b. PV 2, c. PV 3) | [A vs C] 1. $p < 0.05$ (①a~c. C>A ②b. A>C); 2. NS<br>[B vs C] 1. $p < 0.05$ (①b,c. C>B); 2. NS<br>[A vs D] 1,2. ND<br>[B vs D] 1. $p < 0.05$ (①a~c. D>B ②b. B>D); 2. $p < 0.05$ (a,c. D>B)<br>[A vs E] 1. ND; 2. NS<br>[B vs E] 1,2. ND |
| Troy, 2015, USA [28] | RCT | Poliovirus vaccine (HIV patient), 231(A.66/B.66/C.66/D.33) | A. Hollow MNs (IPV: 40% dose, a,b,c); B. Hollow MNs (IPV: 20% dose, a,b,c) | C. IM (IPV: 100% dose, a,b,c) D. IM (IPV: 40% dose, a,b,c) | Deltoid region | 1 (1-day, 1-day) | [4~6 wk after final treatment] 1. Polio immunity; ①Baseline ②1 - month after; 2. Vaccine response; 3. Antibody GMT (a. 40D antigen units of serotype 1, b. 8D antigen units of serotype 2, c. 32D antigen units of serotype 3) | [A vs C] 1,2. NS; 3. ND<br>[B vs C] 1. NS; 2. p=0.01 (c), NS (a,b); 3. ND<br>[A,B vs D] 1,2. NS; 3. ND |
| Levin, 2016, Israel [29] | RCT | Influenza vaccine, 370(A.61/B.61/C.62/D.63/E.63/F.60) | A. Hollow MNs (7.5 μg HA, MJ, a,b,c); B. Hollow MNs (15 μg HA, MJ, a,b,c); C. Hollow MNs (inactivated 15 μg HA, | D. IM (15 μg HA, d); E. IM (45 μg HA, a,b,c); F. IM (15 μg HA with adjuvant, e) | Deltoid region | 1 (1-day, 1-day) | [At 22, 90-days] 1. Immunogenicity (HAI assay) ① Seroconversion rate ② Seroprotection rate ③GMT fold increase (a. A/California/7/2009 vaccine, b. A/Victoria/361/2011, c. B/Hubei-Wujiagang/158/2009, d. Inflexal V™, e. Fluad™, f. Intanza™) | [A vs D] 1. Day 22 ①,②$p < 0.05$ (c) ③$p < 0.05$ (b); Day 90 ①$p < 0.05$ (b) ②$p < 0.05$ (c) ③NS<br>[B vs D]1. Day 22 ①$p < 0.05$ (a~c) ②$p < 0.05$ (c) ③$p < 0.05$ (a~c); Day 90 ①$p < 0.05$ (a) ②,③NS<br>[C vs D]1. NS<br>[A,C vs E] 1. NS<br>[B vs E] 1. ①$p < 0.05$ (a at |

| | | | | | | | | |
|---|---|---|---|---|---|---|---|---|
| | | | SO, f) | | | | | day 22) ②,③NS<br>[A,B,C vs F] 1. NS |
| Vescovo, 2017, Switzerland [30] | RCT* | Rabies vaccine, 64(64/64/64) | A.Hollow MNs (a.b) | B. ID (Mantoux, a,b); C. IM (a,b) | A: Left arm; B: Right arm; C: Non-dominant deltoid region | 3 (28-day, at 0-, 7-, 28-day) | [At 7, 28, 56-day] 1. Pain (VAS) ①Needle insertion ②Products injection; 2. Immunogenicity (RFFIT) (a. Vaccine, b. Saline) | [A vs B] 1. $p < 0.01$ (①A<B ②a. A<B); 2. NS<br>[A vs C] 1. $p < 0.01$ (①A<C ②a. A<C, b. A>C); 2. NS |
| **Insulin delivery** | | | | | | | | |
| Pettis, 2011, USA [31] | RCT(cross-over)* | Type 1 diabetes, 29(29/29) | A. Hollow MNs (IL, 2 min premeal inj.) B. Hollow MNs (RHI, 2 min premeal inj.) C. Hollow MNs (RHI, 17 min premeal inj.) | D. SC (IL, 2 min premeal inj.) E. SC (RHI, 17 min premeal inj.) | Periumbilical abdominal wall | 5 (ND, washout periods of 3~21-days) | [0~240 min after the meal] 1. PD (Super-GL analyzer); ① Insulin PPG at same time ②From postprandial glycemic excursion; 2. PK (Hodges and Lehmann point) ① INS AUC ②$INS_{max}$ ③$tINS_{max}$; 3. Pain (VAS) | [A vs D] 1. ①NS ②$p < 0.05$ ($BG_{min0-4\ h}$); 2. ①$p < 0.05$ (0-0.5,1,1.5,2h) ②,③$p < 0.05$; 3. ND<br>[A vs E] 1. ①ND ②NS; 2. NS; 3. ND<br>[B vs D] 1. ①NS ②$p < 0.05$ ($BG_{2h}$); 2. ①,②NS ③$p < 0.05$; 3. ND<br>[B vs E] 1. ①ND ②NS; 2. NS; 3. ND<br>[C vs D] 1. ①ND ②NS; 2. NS; 3. ND<br>[C vs E] 1. ①$p < 0.05$ ②$p < 0.05$ (BG $AUC_{0-4h}$, $BG_{0-2h}$, $BG_{max,0-4h}$, $BG_{min,0-2h}$, $[BG_{max} – BG_{min}]_{0-4\ h}$); 2. ①$p < 0.05$ (0-0.5,1,1.5,2h) ②,③$p < 0.05$; 3. ND |
| McVey, 2012, USA [32] | RCT(cross-over)* | Type 1 diabetes, 22(22/22) | A. Hollow MNs | B. SC; C. Placebo ID | Periumbilical abdominal wall | 8 (ND, washout periods of 3~14 days) | [0~360 min after treatment] 1. PD (Glucose analyzer) ①time in range ②AUG BG ③BG ④$BG_{mean}$ ⑤$BG_{max}$; 2. PK (Glucose analyzer); 3. Intrasubject and intersubject variability ①PK ②PD; 4. Pain (VAS) ①Insertion ②Infusion | [A vs B] 1. ①NS ②$p < 0.05$ (0-4h, 0-6h) ③$p < 0.05$ (2,4,6h) ④$p < 0.05$ (0-4h, 0-6h) ⑤$p < 0.05$ (0-6h); 2. NS; 3. ①$p < 0.05$ (except $C_{max}$: NS) ②$p < 0.05$; 4. $p < 0.05$ ( ①A>B ②B>A)<br>[A vs C] 1~3. NS; 4. $p < 0.05$ ①NS ②C>A |
| Norman, 2013, USA [33] | RCT(cross-over)* | Type 1 diabetes, 16(16/16) | Hollow MNs | SC | Abdomen | 2 (ND, washout periods of separate days) | [15~240 min after treatment] 1. Pain (VAS) ①Insertion ② Infusion; 2. Onset and offset time; 3. Insulin absorption coefficient; 4. Insulin elimination coefficient; 5. | 1. ①p=0.005 ②NS; 2. $p < 0.05$; 3. $p < 0.05$; 4,5. NS |

| | | | | | | | | |
|---|---|---|---|---|---|---|---|---|
| | | | | | | | Insulin AUC | |
| Rini, 2015, USA [34] | RCT(cross-over)* | Type 1 diabetes mellitus patients, 28(28/28) | Hollow MNs | SC | Abdomen | 2 (multi-wk, washout periods of over 3 days) | [0~4h after treatment] 1. PK endpoints ①$T_{max}$ ② Onset&Offset ③$C_{max}$ ④Insulin AUC; 2. PD endpoints ①AUC-BG ②BG ③$BG_{avg}$ ④$BG_{max}$ 3. Variability ①Intra- ②inter-subject; 4. Perception endpoints ①Self-rated pain (VAS) ②Acceptability and preference | 1. ①~③$p < 0.01$ ④$p < 0.05$ (0-1,1.5h); 2. $p < 0.05$ (①0-1,1.5,2h, ②0-1h, ③0-1.5,2h, ④0-1,1.5,2h); 3. ①$p < 0.05$ ②NS 4. ①ND ②NS |
| Kochba, 2016, Israel [35] | RCT(cross-over* | Type 2 diabetes, 14(a.3/b.6/c.5) (a. Standard meal condition/ b,c. Fasting condition) | Hollow MNs | SC | Lower abdomen (right) | a, b. 1 (4~14days, washout periods of 4~14days) c. 2 (4~14days, washout periods of 4~14days) | [0~6h after treatment] 1. PK ①$T_{max}$, ②$C_{max}$ ③Onset ④ AUC; 2. PD ①Insulin AUC ② Glucose AUC; 3. The relative bioavailability; 4. Pain (VAS) ① Insertion ②Infusion | [Standard meal and fasting condition] 1. ①,③$p < 0.05$ ②,④NS; 2. ①$p < 0.05$ ② ND 3. NS; 4. ①NS ②$p <$ 0.05 (Int<Con) [Only standard meal condition] 1~3. ND; 4. ①NS ②$p < 0.05$ (Int<Con) [Only fasting condition] ①, ③$p < 0.05$ ②,④NS; 2. ①$p$ < 0.05 ②$p < 0.05$ (4-6h); 3. NS; 4. ①NS ②$p < 0.05$ (Int<Con) |
| **Others** | | | | | | | | |
| Cosman, 2010, USA [36] | RCT | Postmenopausal osteoporosis 165(A.34/B.32/C.33/D.33/E.33) | A. MAP (20μg TPDP); B. MAP (30μg TPDP); C. MAP (40μg TPDP) | D. SC (Injection pen); E. Placebo patch | A~C,E. The lateral abdomen (alternating left and right sides on a daily basis); D. The abdomen or thigh | 28 (28-day, daily) | [6-mon after final treatment] 1. BMD (DXA) ①lumbar spine; ② left total hip; ③femoral neck; ④left forearm; 2. Bone formation marker ①serum P1NP; ②serum CTX; 3. Pharmacokinetic ①AUC; ②$C_{max}$ | [A,B,C vs D] 1. NS; 2,3. ND<br>[A,B vs E] 1. ①$p < 0.001$ ② NS ③,④NS; 2,3. ND [C vs E] 1. ①$p < 0.001$ ②$p$ < 0.05 ③,④NS; 2,3. ND |
| Daddona, 2011, USA [37] | RCT | Osteoporosis, 165(A.33/B.33/C.33/D.33/E.33) | A. MAP (PTH 20μg); B. MAP (PTH 30μg); C. MAP (PTH 40μg) | D. SC (Injection pen); E. Placebo patch | Abdomen, thigh or arm | About 180 (6-mon, daily) | [At 6-mon] 1. Estimates of AUC; 2. Estimates of $C_{max}$; 3. BMD ①lumbar spine (after 6-mon); ②left total hip (after 6-mon) | [A,B vs D] 1,2. ND; 3. ①,② NS [C vs D] 1,2. ND; 3. ①NS ② $p < 0.05$<br>[A,B vs E] 1,2. ND; 3. ①$p <$ 0.05 ② NS [C vs E] 1,2. ND; 3. ①$p <$ 0.05 ②$p < 0.05$ |

| | | | | | | | | |
|---|---|---|---|---|---|---|---|---|
| Spierings, 2018, USA [38] | RCT | Migraine, 365(A.90/B.92/C.92/D.91) | A. MAP (zolmitriptan 1 mg); B. MAP (zolmitriptan 1.9 mg); C. MAP (two zolmitriptan 1.9 mg) | D. Placebo patch | The upper arm | A,B,D. 1 (48-h, 1-day); C. 2 (48-h, 1-day) | [2~48h after final treatment] 1. Pain freedom rate (①after 2h; ② 2-24h; ③2-48h); 2. Pain relief rate ( ①2-24h; ②2-48h); 3. MBS freedom rate (after 2h); 4. Patient rate (after 2h) ①Photophobia-free; ②Phonophobia-free; ③Nausea-free; ④Took rescue medication | [A,B vs D] 1,2. $p < 0.05$; 3. NS; 4. $p < 0.05$ (①), NS (②~④) [C vs D] 1,2. $p < 0.05$; 3. $p < 0.05$; 4. $p < 0.05$ (①,③), NS (②,④) |

AHA, Alpha hydroxyl acid; ALA, aminolevulinic acid; AUC, area under the curve; $AUC_{10-360}$, AUC from 10 to 360 min; BG, blood glucose; BMD, bone mineral density; bOPV, bivalent oral poliovirus vaccine; BoNT/A, botulinum toxin A; CCT, clinical control trial; Con, control group; DXAM dual-energy x-ray absorptiometry; CTX, C-terminal cross-linked telopeptide of type 1 collagen; ECCA, echelle d'evaluation clinique des cicatrices d'acne; ED, epidermal; EGF, epidermal growth factor; f-IPV, fractional dose inactivated poliovirus vaccine; GIRs, glucose infusion rates; GMT, Geometric mean titers; GPS, Global photodamage score; HA, haemagglutination; HAI, haemagglutination inhibition; h, hour; HDSS, hyperhidrosis disease severity scale; HI, haemagglutination inhibition; HIV, human immunodeficiency virus; ID, intradermal injection; imp, improvement; IL, insulin lispro; IM, intramuscular injection; inj., Injection; Int, intervention group; IPV, inactivated poliovirus vaccine; ITA°, individual typology angle; MAL, methyl 5-aminolevulinic acid; MAP, microneedle array patch; MBS, bothersome migraine symptom; MI, melanin index; mom, month; MJ, micronejet; MN, microneedle; MRF; microneedle radiofrequency; MTS, microneedle therapy system; ND, not described; NS, not significant; OPV, oral poliovirus vaccine; PGA, physician's global assessment; PaGA, patient's global assessment; PD, pharmacodynamic; PK, pharmacokinetics; POSAS, patient and observer scar assessment; PPG, pharmacodynamic postprandial glycemia; PTH, parathyroid hormone; PV, poliovirus type; P1NP, procollagen type 1 n-terminal propeptide; RCT, randomized control trial; RHI, regular human insulin; SC, Subcutaneous injection; SO, soulvia; TEWL, transepidermal water loss measurement; $tGIR_{max}$, time to maximum GIR; TIV, trivalent inactivated influenza vaccine; tOPV, trivalent oral poliovirus vaccine; TPTD, teriparatide; VAS, visual analog scale; wk, week; VNA, virus neutralizing antibody; *, These studies shown in which both intervention and control were applied to different areas of a patient (e.g., treatment and control interventions were applied to the left and right faces of one subject, respectively).

**Table S3.** Type of adverse events reported in each study.

| Disease. | Author, Year | Disorder | Intervention | Control | Type of AEs | | Grade |
|---|---|---|---|---|---|---|---|
| | | | | | Local | Systemic | |
| Scar | Khater, 2016 [1] | Striae distensae | MTS | $CO_2$ laser | Int&Con) Erythema, PIH | None | Grade 1 |
| | Busch, 2018[2] | Hypertrophic burn scar | MTS | No treatment | Int&Con) Pruritus | None | Grade1 |
| | Ryu, 2013 [3] | Striae distensae | MRF | $CO_2$ laser | Int&Con) PIH, Pruritus, Int) Pain | None | Grade 1 |
| | Chae, 2014 [4] | Atrophic acne scar | MRF | Er:Glass fractional laser | Int&Con) Erythema, Edema, Dryness<br>Con) PIH, Induce acne vulagris | None | Grade 1 |
| Facial wrinkle | Jeon, 2013 [5] | Periorbital static wrinkles | MRF | BoNT/A | Int) Bleeding, Bruising, PIH, Pain | None | Grade 1 |
| | Lu, 2017 [6] | Periorbital/nasolabial wrinkles | MRF | Superficial insertion | Int&Con) Crusting, Erythema, Petechia, Edema | None | Grade 1 |
| | Lee, 2016 [7] | Periorbital wrinkles | MAP | Placebo patch | ND | ND | ND |
| | Hong, 2018 [8] | Periorbital/nasolabial wrinkles | MAP | No-treatment | None | None | None |
| Skin care | Kim, 2009 [9] | Facial skin condition | MTS | Alpha hydroxyl acid peeling | Int) Stinging, Flush, Flaky skin, Burn<br>Con) Flush, Flaky skin, Burn, Stinging on eyes, Itching | None | Grade 1 |
| | Choi, 2011 [10] | Facial skin condition | MTS | High frequency treatment | ND | ND | ND |
| | Kim, 2016 [11] | Facial hyperpigmentation | MAP | Placebo patch | Int&Con) Erythema | None | Grade 1 |
| | Park, 2017 [12] | Fitzpatrick skin type | MAP | Whitening essence | None | None | None |
| Alopecia | Yoo, 2010 [13] | Alopecia areata | MTS | MAL cream | Int&Con) Erythema<br>Int) Pain | None | Grade 1 |
| | Bao, 2017 [14] | Androgenetic alopecia | AMTS | Minoxidil (topical) | Int) Eczema, Increased Scurf, Auricular Lymph node, Enlargement of cervical or posterior, Pain<br>Con) Seborrheic dermatitis, Itching, Eczema | Int) Infection | Grade 1 |

| | | | | | | | |
|---|---|---|---|---|---|---|---|
| Actinic keratoses | Petukhova, 2017 [15] | Actinic keratoses | MTS | Placebo roller | Int&Con) Pain | ND | Grade 1 |
| | Spencer, 2016 [16] | Actinic keratoses | MTS | ALA | ND | ND | ND |
| Skin (others) | Shin, 2012 [17] | Acne | MRF | $CO_2$ laser | Int&Con) Erythema, Oedema, Pain<br>Con) Hyperpigmentation | None | Grade 1 |
| | Fatemi Naeini, 2015 [18] | Axillary hyperhidrosis | MRF | Placebo MRF (standby mode) | Int&Con) Swelling, Redness, Erythema, Pinpoint bleeding, PIH, Tingling, Numbness | None | Grade 1 |
| | Ryu, 2018 [19] | Multiple warts | MAP | Cryotherapy | Int&Con) Hyperpigmentation, Scar, Nail dystrophy, Delayed response, Pain<br>Int) Erythema | Int&Con) Infection | Grade 1 |
| Vaccine delivery | Hirobe, 2015 [20] | Influenza vaccine | MAP | SC | Int&Con) Erythema, Purpura, Pigmentation, Induration | Int&Con) Fever | Grade 1 |
| | Rouphael, 2017 [21] | Influenza vaccine | MAP | 1. IM<br>2. Placebo patch | Int&Con) Pain<br>Int) Induration, Erythema, Tenderness, Pruritis<br>Con1) Induration, Tenderness, Pruritis<br>Con2) Tenderness, Pruritis | Int&Con) Fatigue, Myalgia, Shivering or shaking body movements, Nausea, Headache, Arthralgia, Malaise<br>Int) Fever, Sweating<br>Con1) Sweating | Grade 1 |
| | Fernando, 2018 [22] | Influenza vaccine | MAP | 1. IM<br>2. Placebo patch<br>3. Placebo IM | Int) Application site pruritus, Axillary pain, Sinusitis, Oropharyngeal pain<br>Con) Oropharyngeal pain, Throat irritation | Int) Headache, Lymph-adenopathy, viral URTI, Myalgia<br>Con) Headache, Fatigue, Upper respiratory tract infection (URTI) | Grade 1 |
| | Damme, 2009 [23] | Influenza vaccine | Hollow MN s | IM | Int&Con) Erythema, Swelling, Induration, Pain<br>Int) Discoloration, Pruritus, Desquamation | Int&Con) Headache, Malaise, Myalgia | Grade 1 |
| | Laurent, 2010 [24] | Influenza vaccine | Hollow MNs | 1. IM<br>2. ED | Int&Con) Erythema, Induration, Itching, Swelling<br>Int&Con1) Pain<br>Con)The deltoid muscle pain | Int) Myalgia, Headache, Flu-like symptoms | Grade 1 |
| | Frenck, 2011 [25] | Influenza vaccine | 1. Hollow MNs (6μg HA/strain) | 1. IM<br>2. ID | Int&Con) Itching, Redness, Swelling, Pain | Int&Con) Headache, Fever<br>Int2) Acute disseminated encephalomyelitis* | All - Grade 1 |

| | | | | | | | |
|---|---|---|---|---|---|---|---|
| | | | 2. Hollow MNs (9µg HA/strain) | (Mantoux) | | | * - Grade 3 |
| | Levin, 2014 [26] | Influenza vaccine | Hollow MNs | 1. ID (Mantoux)<br>2. IM | Int&Con) Post-vaccination pain, Induration, Erythema | ND | Grade 1 |
| | Anand, 2015 [27] | Poliovirus vaccine | Hollow MNs | 1. tOPV (Oral)<br>2. bOPV (Oral)<br>3. IPV (IM) | None | Int) Allergic rhinitis, Allergic conjunctivitis, Chicken pox, Common cold, Measles, Tinea capitis, Acute ottis media, Fever with no infectious focus, Fever probably related to pentavalent, Infective dermatitis, Scabies, Diarrhoea*, Pneumonia**, Infantile seizures**, Sudden infant death syndrome**<br>Con1) Sudden infant death syndrome, Acute gastroenteritis, Allergic conjunctivitis, Boil, Common cold, Fever, Diarrhoe*, Pneumonia*<br>Con2) Common cold, Fever, Oral candidiasis, Skin rash, Acute gastroenteritis*, Pneumonia*<br>Con3) Common cold, Fever, Oral candidiasis, Diarrhoea*, Meningits* | Grade 1<br>* - Grade 3<br>** - Grade 5 |
| | Troy, 2015 [28] | Influenza vaccine | Hollow MNs | IM | Int&Con) Swelling, Tederness, Redness<br>Int) Itching | Int&Con) Rash, Fever | Grade1 |
| | Levin, 2016 [29] | Influenza vaccine | 1. Hollow MNs (7.5 µg HA, MJ)<br>2. Hollow MNs (15 µg HA, MJ)<br>3. Hollow MNs (inactivated 15 µg HA, SO) | 1. IM (15 µg HA)<br>2. IM (45 µg HA)<br>3. IM (15 µg HA with adjuvant) | Int&Con) Erythema, Ecchymosis, Induration<br>Int2) Polymyalgia rheumatic* | Int&Con) Fever, Shivering, Malaise<br>Con3) Death** | Grade 1<br>* - Grade 3<br>** - Grade 5 |
| | Vescovo, 2017 [30] | The rabies vaccine delivery | Hollow MNs | 1. ID (Mantoux)<br>2. IM | Int & Con) Redness, Pruritus, Pain | Int&Con) Headache, Gastrointestinal disorder, Asthenia, Dizziness, Malaise, Nausea, Myalgia | Grade 1 |
| Insulin delivery | Pettis, 2011 [31] | Type 1 diabetes | Hollow MNs | SC | Int&Con) Pain<br>Int) Edema | Int&Con) Headache, Diarrhea, Hypoglycemic episodes | Grade 1 |
| | McVey, 2012 [32] | Type 1 diabetes | Hollow MNs | 1. SC<br>2. Placebo MNs | Int&Con) Erythema, Edema, Pain | Int&Con) Headache | Grade 1 |
| | Norman, 2013 [33] | Type 1 diabetes | Hollow MNs | SC | Int&Con) Blebs, Pain | None | Grade 1 |
| | Rini, 2015 [34] | Type 1 diabetes mellitus patients | Hollow MNs | SC | Int&Con) Erythema, Edema, Pain | Int&Con) Headache, Dizziness, Nausea, Vomiting | Grade 1 |
| | Kochba, | Type 2 diabetes | Hollow MNs | SC | Int&Con) Pain | Int&Con) Hypoglycemia | Grade 1 |

| | | | | | | | |
|---|---|---|---|---|---|---|---|
| | 2016 [35] | | | | | | |
| Others | Cosman, 2010 [36] | Postmenopausal | MAP | 1. SC<br>2. Placebo patch | Int&Con) Erythema | Int&Con) Arthralgia/pain in extremities, Dizziness, Headache, Nausea, Serum calcium elevation, Hypercalcemia*, Hypercalciuria*<br>Int) Urine/creatinine ratio increased*, Nephrolithiasis*<br>Con) Crystal urine, Hypotension, Syncope, Blood calcium decreased* | All – Grade 1<br>* - Grade 2 |
| | Daddona, 2011 [37] | Osteoporosis | MAP | 1. Injection pen<br>2. Placebo patch | Int & Con 2) Erythema | None | Grade 1 |
| | Spierings, 2018 [38] | Migraine | 1. MAP (1mg zolmitriptan)<br>2. MAP (1.9mg zolmitriptan)<br>3. MAP (3.8mg zolmitriptan) | Placebo patch | Int&Con) Erythema, Bruise, Pain, Swelling/edema<br>Int) Hemorrhage, Redness<br>Int 3) Bruise*, Pain* | Int&Con) Paresthesia<br>Int) Dizziness, Muscle tightness, Nausea<br>Int 3) Muscle tightness* | All - Grade 1<br>* - Grade 2 |

*, not grade 1 AE; AE, Adverse event; AMTS, auto microneedle therapy system; bOPV, bivalent oral poliovirus vaccines; Con, control group; ED, epidermal; HA, haemagglutination; HIV, human immunodeficiency virus; ID, intradermal; Int, intervention group; IM, intramuscular injection; IPV, inactivated poliovirus vaccine; MAL, methyl 5-aminolevulinic acid; MAP, microneedle array patch; MJ, micronjet; MTS, microneedle therapy system; MN, microneedle; MRF, microneedle radiofrequency; NA, Not applicable; None, No adverse events; PIH, post inflammatory hyperpigmentation; SC, subcutaneous injection; SO, soulvia; tOPV. trivalent oral poliovirus vaccines; TPTD, teriparatide.

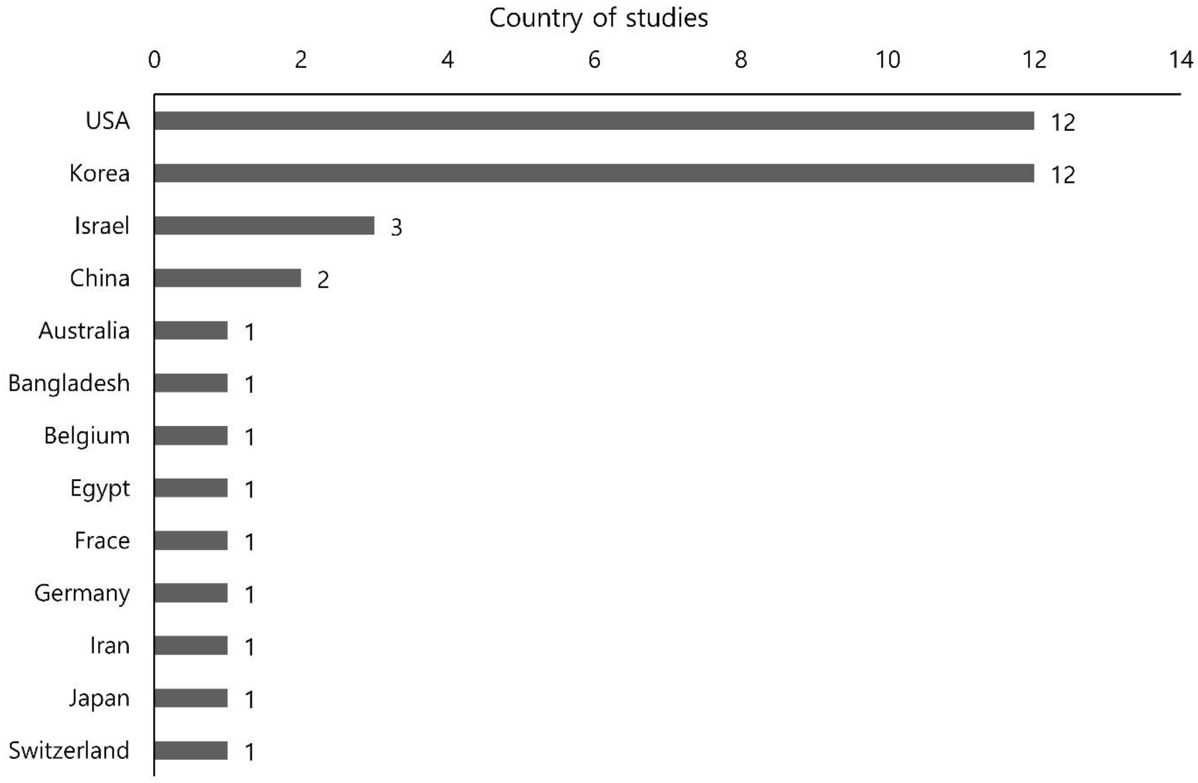


**Figure S1.** Number of studies included by country.

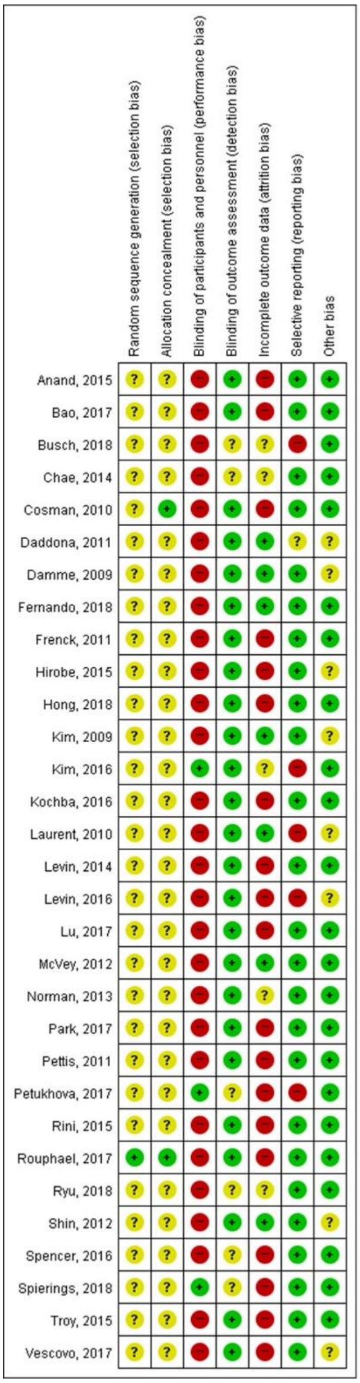


**Figure S2.** Risk of bias summary of randomized controlled trials (RCT).

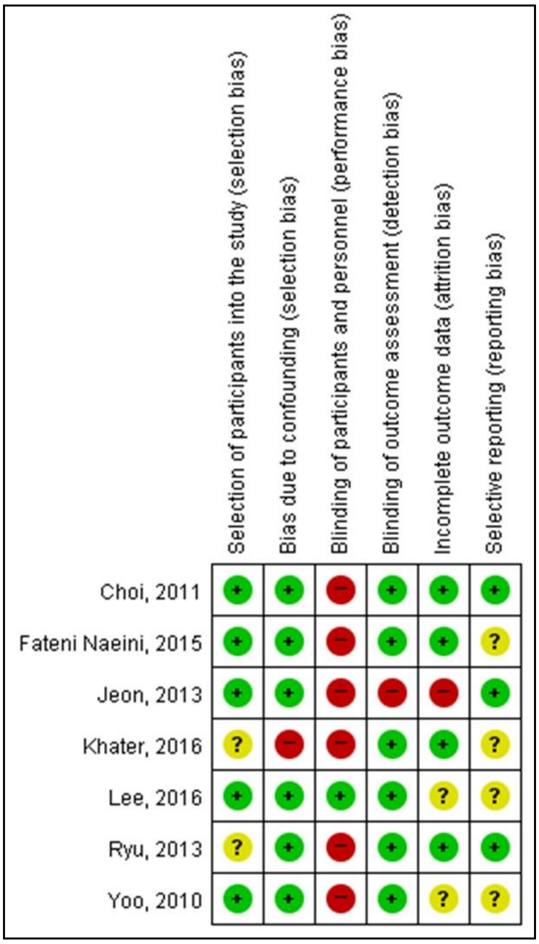


**Figure S3.** Risk of bias summary of controlled clinical trials (CCTs).

## References


1 Khater, M.H.; Khattab, F.M.; Abdelhaleem, M.R. Treatment of striae distensae with needling therapy versus CO2 fractional laser. *J. Cosmet. Laser Ther.* **2016,** *18* (2), 75-79, doi:10.3109/14764172.2015.1063665.

2 Busch, K.-H.; Aliu, A.; Walezko, N.; Aust, M. Medical Needling: Effect on Moisture and Transepidermal Water Loss of Mature Hypertrophic Burn Scars. *Cureus* **2018,** *10* (3), doi:10.7759/cureus.2365.

3 Ryu, H.W.; Kim, S.A.; Jung, H.R.; Ryoo, Y.W.; Lee, K.S.; Cho, J.W. Clinical Improvement of Striae Distensae in K orean Patients Using a Combination of Fractionated Microneedle Radiofrequency and Fractional Carbon Dioxide Laser. *Derm. Surg* **2013,** *39* (10), 1452-1458, doi:10.1111/dsu.12268.

4 Chae, W.S.; Seong, J.Y.; Jung, H.N.; Kong, S.H.; Kim, M.H.; Suh, H.S.; Choi, Y.S. Comparative study on efficacy and safety of 1550 nm Er: Glass fractional laser and fractional radiofrequency microneedle device for facial atrophic acne scar. *J. Cosmet. Dermatol.* **2015,** *14* (2), 100-106, doi:10.1111/jocd.12139.

5 Jeon, I.K.; Chang, S.E.; Park, G.-H.; Roh, M.R. Comparison of microneedle fractional radiofrequency therapy with intradermal botulinum toxin a injection for periorbital rejuvenation. *Dermatology* **2013,** *227* (4), 367-372, doi:10.1159/000356162.

6 Lu, W.; Wu, P.; Zhang, Z.; Chen, J.; Chen, X.; Ewelina, B. Curative effects of microneedle fractional radiofrequency system on skin laxity in Asian patients: A prospective, double-blind, randomized, controlled face-split study. *J. Cosmet. Laser Ther.* **2017,** *19* (2), 83-88, doi:10.1080/14764172.2016.1256485.

7 Lee, C.; Yang, H.; Kim, S.; Kim, M.; Kang, H.; Kim, N.; An, S.; Koh, J.; Jung, H. Evaluation of the anti-wrinkle effect of an ascorbic acid-loaded dissolving microneedle patch via a double-blind, placebo-controlled clinical study. *Int. J. Cosmet. Sci.* **2016**, *38*, 375–381, doi:10.1111/ics.12299.

8 Hong, J.Y.; Ko, E.J.; Choi, S.Y.; Li, K.; Kim, A.R.; Park, J.O.; Kim, B.J. Efficacy and safety of a novel, soluble microneedle patch for the improvement of facial wrinkle. *J. Cosmet. Dermatol.* **2018**, *17*, 235–241, doi:10.1111/jocd.12426.

9 Kim, Y.K.; Jang, K.J.; Li, S.H.; An, S.K.; Choe, T.B. The Effect of Microneedle Therapy System and Alpha Hydroxy Acid Peeling on the Pores and the Hyperpigmentation of Men's Skin. *Kor J Aesthet Cosmetol* **2009,** *7* (1), 69-79.

10 Choi, Y.E. The effect of microneedle therapy system and high frequency to improve the facial skin conditions for mid-aged women. Master's Thesis, Sungshin Wo Men's University, Korea, 2011; pp. 1–93 **2011**.

11 Kim, S.; Yang, H.; Kim, M.; Baek, J.H.; Kim, S.J.; An, S.M.; Koh, J.S.; Seo, R.; Jung, H. 4-n-butylresorcinol dissolving microneedle patch for skin depigmentation: a randomized, double-blind, placebo-controlled trial. *J. Cosmet. Dermatol.* **2016**, *15*, 16–23, doi:10.1111/jocd.12178.

12 Park, K.Y.; Kwon, H.J.; Lee, C.; Kim, D.; Yoon, J.J.; Kim, M.N.; Kim, B.J. Efficacy and safety of a new microneedle patch for skin brightening: A Randomized, split-face, single-blind study. *J. Cosmet. Dermatol.* **2017**, *16*, 382–387, doi:10.1111/jocd.12354.

13 Yoo, K.H.; Lee, J.W.; Li, K.; Kim, B.J.; Kim, M.N. Photodynamic therapy with methyl 5-aminolevulinate acid might be ineffective in recalcitrant alopecia totalis regardless of using a microneedle roller to increase skin penetration. *Derm. Surg.* **2010**, *36*, 618–622, doi:10.1111/j.1524-4725.2010.01515.x.

14 Bao, L.L.; Gong, L.; Guo, M.; Liu, T.; Shi, A.; Zong, H.; Xu, X.; Chen, H.; Gao, X.; Li, Y. Randomized trial of electrodynamic microneedle combined with 5% minoxidil topical solution for the treatment of Chinese male Androgenetic alopecia. *J. Cosmet.* **2017**, *22*, 1–7, doi:10.1080/14764172.2017.1376094.

15 Petukhova, T.A.; Hassoun, L.A.; Foolad, N.; Barath, M.; Sivamani, R.K. Effect of expedited microneedle-assisted photodynamic therapy for field treatment of actinic keratoses: a randomized clinical trial. *JAMA dermatology* **2017,** *153* (7), 637-643, doi:10.1001/jamadermatol.2017.0849.

16 Spencer, J.M.; Freeman, S.A. Microneedling prior to Levulan PDT for the treatment of actinic keratoses: a split-face, blinded trial. *J Drugs Dermatol* **2016,** *15* (9), 1072-1074, doi:10.3410/f.726717627.793536654.

17 Shin, J.U.; Lee, S.H.; Jung, J.Y.; Lee, J.H. A split-face comparison of a fractional microneedle radiofrequency device and fractional carbon dioxide laser therapy in acne patients. *J. Cosmet.* **2012**, *14*, 212–217, doi:10.3109/14764172.2012.720023.

18 Fatemi Naeini, F.; Abtahi-Naeini, B.; Pourazizi, M.; Nilforoushzadeh, M.A.; Mirmohammadkhani, M. Fractionated microneedle radiofrequency for treatment of primary axillary hyperhidrosis: a sham control study. *Australasian. J. Dermatol.* **2015**, *56*, 279–284, doi:10.1111/ajd.12260

19 Ryu, H.R.; Jeong, H.-R.; Seon-Woo, H.-S.; Kim, J.S.; Lee, S.K.; Kim, H.J.; Baek, J.O.; Park, J.-H.; Roh, J.Y. Efficacy of a bleomycin microneedle patch for the treatment of warts. *Drug Deliv. Transl. Res.* **2018**, *8*, 273–280, doi:10.1007/s13346-017-0458-4.

20 Hirobe, S.; Azukizawa, H.; Hanafusa, T.; Matsuo, K.; Quan, Y.S.; Kamiyama, F.; Katayama, I.; Okada, N.; Nakagawa, S. Clinical study and stability assessment of a novel transcutaneous influenza vaccination using a dissolving microneedle patch. *Biomaterials* **2015,** *57*, 50-58, doi:10.1016/j.biomaterials.2015.04.007.

21 Rouphael, N.G.; Paine, M.; Mosley, R.; Henry, S.; McAllister, D.V.; Kalluri, H.; Pewin, W.; Frew, P.M.; Yu, T.; Thornburg, N.J. The safety, immunogenicity, and acceptability of inactivated influenza vaccine delivered by microneedle patch (TIV-MNP 2015): a randomised, partly blinded, placebo-controlled, phase 1 trial. *The Lancet* **2017,** *390* (10095), 649-658, doi:10.1016/S0140-6736(17)30575-5.

22 Fernando, G.J.; Hickling, J.; Flores, C.M.J.; Griffin, P.; Anderson, C.D.; Skinner, S.R.; Davies, C.; Witham, K.; Pryor, M.; Bodle, J. Safety, tolerability, acceptability and immunogenicity of an influenza vaccine delivered to human skin by a novel high-density microprojection array patch (Nanopatch™). *Vaccine* **2018,** *36* (26), 3779-3788, doi:10.1016/j.vaccine.2018.05.053.

23 Van Damme, P.; Oosterhuis-Kafeja, F.; Van der Wielen, M.; Almagor, Y.; Sharon, O.; Levin, Y.J.V. Safety and efficacy of a novel microneedle device for dose sparing intradermal influenza vaccination in healthy adults. **2009,** *27* (3), 454-459, doi:10.1016/j.vaccine.2008.10.077.

24 Laurent, P.E.; Bourhy, H.; Fantino, M.; Alchas, P.; Mikszta, J.A. Safety and efficacy of novel dermal and epidermal microneedle delivery systems for rabies vaccination in healthy adults. *Vaccine* **2010,** *28* (36), 5850-5856, doi:10.1016/j.vaccine.2010.06.062.

25 Frenck Jr, R.W.; Belshe, R.; Brady, R.C.; Winokur, P.L.; Campbell, J.D.; Treanor, J.; Hay, C.M.; Dekker, C.L.; Walter Jr, E.B.; Cate, T.R. Comparison of the immunogenicity and safety of a split-virion, inactivated,

trivalent influenza vaccine (Fluzone®) administered by intradermal and intramuscular route in healthy adults. *Vaccine* **2011,** *29* (34), 5666-5674, doi:10.1016/j.vaccine.2011.06.010.
26 Levin, Y.; Kochba, E.; Kenney, R. Clinical evaluation of a novel microneedle device for intradermal delivery of an influenza vaccine: are all delivery methods the same? *Vaccine* **2014,** *32* (34), 4249-4252, doi:10.1016/j.vaccine.2014.03.024.
27 Anand, A.; Zaman, K.; Estívariz, C.F.; Yunus, M.; Gary, H.E.; Weldon, W.C.; Bari, T.I.; Oberste, M.S.; Wassilak, S.G.; Luby, S.P. Early priming with inactivated poliovirus vaccine (IPV) and intradermal fractional dose IPV administered by a microneedle device: A randomized controlled trial. *Vaccine* **2015,** *33* (48), 6816-6822, doi:10.1016/j.vaccine.2015.09.039.
28 Troy, S.B.; Kouiavskaia, D.; Siik, J.; Kochba, E.; Beydoun, H.; Mirochnitchenko, O.; Levin, Y.; Khardori, N.; Chumakov, K.; Maldonado, Y. Comparison of the immunogenicity of various booster doses of inactivated polio vaccine delivered intradermally versus intramuscularly to HIV-infected adults. *The Journal of infectious diseases* **2015,** *211* (12), 1969-1976, doi:10.1093/infdis/jiu841.
29 Levin, Y.; Kochba, E.; Shukarev, G.; Rusch, S.; Herrera-Taracena, G.; van Damme, P.J.V. A phase 1, open-label, randomized study to compare the immunogenicity and safety of different administration routes and doses of virosomal influenza vaccine in elderly. **2016,** *34* (44), 5262-5272, doi:10.1016/j.vaccine.2016.09.008.
30 Vescovo, P.; Rettby, N.; Ramaniraka, N.; Liberman, J.; Hart, K.; Cachemaille, A.; Piveteau, L.-D.; Zanoni, R.; Bart, P.-A.; Pantaleo, G. Safety, tolerability and efficacy of intradermal rabies immunization with DebioJect™. *Vaccine* **2017,** *35* (14), 1782-1788, doi:10.1016/j.vaccine.2016.09.069.
31 Pettis, R.J.; Hirsch, L.; Kapitza, C.; Nosek, L.; Hövelmann, U.; Kurth, H.-J.; Sutter, D.E.; Harvey, N.G.; Heinemann, L. Microneedle-based intradermal versus subcutaneous administration of regular human insulin or insulin lispro: pharmacokinetics and postprandial glycemic excursions in patients with type 1 diabetes. *Diabetes Technol.* **2011,** *13* (4), 443-450, doi:10.1089/dia.2010.0183.
32 McVey, E.; Hirsch, L.; Sutter, D.E.; Kapitza, C.; Dellweg, S.; Clair, J.; Rebrin, K.; Judge, K.; Pettis, R.J., Pharmacokinetics and postprandial glycemic excursions following insulin lispro delivered by intradermal microneedle or subcutaneous infusion. *J. Diabetes Sci. Technol.* **2012**, *6*, 1–12, doi:10.1177/193229681200600403.
33 Norman, J.J.; Brown, M.R.; Raviele, N.A.; Prausnitz, M.R.; Felner, E.I. Faster pharmacokinetics and increased patient acceptance of intradermal insulin delivery using a single hollow microneedle in children and adolescents with type 1 diabetes. *Pediatric diabetes* **2013,** *14* (6), 459-465, doi:10.1111/pedi.12031.
34 Rini, C.J.; McVey, E.; Sutter, D.; Keith, S.; Kurth, H.-J.; Nosek, L.; Kapitza, C.; Rebrin, K.; Hirsch, L.; Pettis, R.J. Intradermal insulin infusion achieves faster insulin action than subcutaneous infusion for 3-day wear. *Drug Deliv. Transl. Res.* **2015**, *5*, 332–345, doi:10.1007/s13346-015-0239-x.
35 Kochba, E.; Levin, Y.; Raz, I.; Cahn, A. Improved insulin pharmacokinetics using a novel microneedle device for intradermal delivery in patients with type 2 diabetes. *Diabetes Technol. Ther.* **2016**, *18*, 525–531, doi:10.1089/dia.2016.0156.
36 Cosman, F.; Lane, N.E.; Bolognese, M.A.; Zanchetta, J.R.; Garcia-Hernandez, P.A.; Sees, K.; Matriano, J.A.; Gaumer, K.; Daddona, P.E. Effect of transdermal teriparatide administration on bone mineral density in postmenopausal women. *J. Clin. Endocrinol. Metab.* **2010**, *95*, 151–158, doi:10.1210/jc.2009-0358.
37 Daddona, P.E.; Matriano, J.A.; Mandema, J.; Maa, Y.F. Parathyroid hormone (1-34)-coated microneedle patch system: clinical pharmacokinetics and pharmacodynamics for treatment of osteoporosis. *Pharm. Res.* **2011**, *28*, 159–165, doi:10.1007/s11095-010-0192-9
38 Spierings, E.L.; Brandes, J.L.; Kudrow, D.B.; Weintraub, J.; Schmidt, P.C.; Kellerman, D.J.; Tepper, S.J. Randomized, double-blind, placebo-controlled, parallel-group, multi-center study of the safety and efficacy of ADAM zolmitriptan for the acute treatment of migraine. *Cephalalgia* **2018,** *38* (2), 215-224, doi:10.1177/0333102417737765.
